# Evidence for a cytoplasmic pool of ribosome-free mRNAs encoding inner membrane proteins in *Escherichia coli*

**DOI:** 10.1371/journal.pone.0183862

**Published:** 2017-08-25

**Authors:** Daniel Benhalevy, Ido Biran, Elena S. Bochkareva, Rotem Sorek, Eitan Bibi

**Affiliations:** 1 Department of Biological Chemistry, Weizmann Institute of Science, Rehovot, Israel; 2 Department of Molecular Genetics, Weizmann Institute of Science, Rehovot, Israel; Centre National de la Recherche Scientifique, Aix-Marseille Université, FRANCE

## Abstract

Translation-independent mRNA localization represents an emerging concept in cell biology. In *Escherichia coli*, mRNAs encoding integral membrane proteins (MPRs) are targeted to the membrane where they are translated by membrane associated ribosomes and the produced proteins are inserted into the membrane co-translationally. In order to better understand aspects of the biogenesis and localization of MPRs, we investigated their subcellular distribution using cell fractionation, RNA-seq and qPCR. The results show that MPRs are overrepresented in the membrane fraction, as expected, and depletion of the signal recognition particle-receptor, FtsY reduced the amounts of all mRNAs on the membrane. Surprisingly, however, MPRs were also found relatively abundant in the soluble ribosome-free fraction and their amount in this fraction is increased upon overexpression of CspE, which was recently shown to interact with MPRs. CspE also conferred a positive effect on the membrane-expression of integral membrane proteins. We discuss the possibility that the effects of CspE overexpression may link the intriguing subcellular localization of MPRs to the cytosolic ribosome-free fraction with their translation into membrane proteins and that the ribosome-free pool of MPRs may represent a stage during their targeting to the membrane, which precedes translation.

## Introduction

Integral membrane proteins (IMPs) are usually translated by membrane bound ribosomes. The question how mRNAs encoding membrane proteins (MPRs) reach the membrane remained controversial: Does targeting occur during IMPs translation together with the translating ribosomes [1–4] or in a translation-independent manner [5–9], or via a more complex combination of these two pathways. Translation-independent targeting requires specific recognition and handling of MPRs that differ from other mRNAs, such as those encoding cytoplasmic proteins (CPRs). Generally, for selective subcellular localization, mRNAs utilize various protein-interaction determinants (structural, sequence specific, or nonspecific) [10], mostly in 3’ untranslated regions. In this regard, *E*. *coli* and other bacteria represent an interesting case because unlike in eukaryotes [11] the prokaryotic mRNAs usually contain very little regulatory information in their 3’ UTRs [12, 13]. Previously, we proposed that MPRs in *E*. *coli*, and possibly also elsewhere, might be selectively recognized through features derived from their high uracil content in long segments throughout their coding sequence (~60 nucleotide-long) [14]. To investigate this hypothesis, we previously searched for uracil-rich RNA-binding proteins [15]. These studies led to the identification a highly specific interaction that takes place between transcripts that mimic MPRs and the cold shock proteins CspE and CspC, which are normally expressed under physiological conditions. The specific interaction with CspE occurred *in vivo* not only with the model uracil-rich transcripts but also with endogenous MPRs [15]. Here, towards better understanding of the biogenesis of MPRs and their interactions with cold shock proteins, we utilized biochemical fractionation to investigate the subcellular distribution of mRNAs in *Escherichia coli* with emphasis on MPRs and the role of CspE. Mapping the RNA content of cellular fractions by next generation sequencing (NGS) offers an estimation for the overall subcellular location of MPRs and CPRs in this bacterium. Our analyses confirmed the notion that MPRs are more abundant on the membrane as shown recently by super-resolution imaging of fixed and permeabilized *E*. *coli* cells [4]. Counter-intuitively, however, a large portion of the MPRs was found in the ribosome-free soluble fraction. We hypothesize that the pool of MPRs in the ribosome-free fraction may represent an earlier stage during their targeting to the membrane. Finally, our results revealed that overexpression of the cold shock protein CspE specifically increased the MPRs pool in the ribosome-free fraction and their amount on the membrane and positively affected their translation into integral membrane proteins.

## Results

### Cell fractionation, qPCR, and high-throughput sequencing of mRNAs in wild-type *E*. *coli*

**Fig 1:**
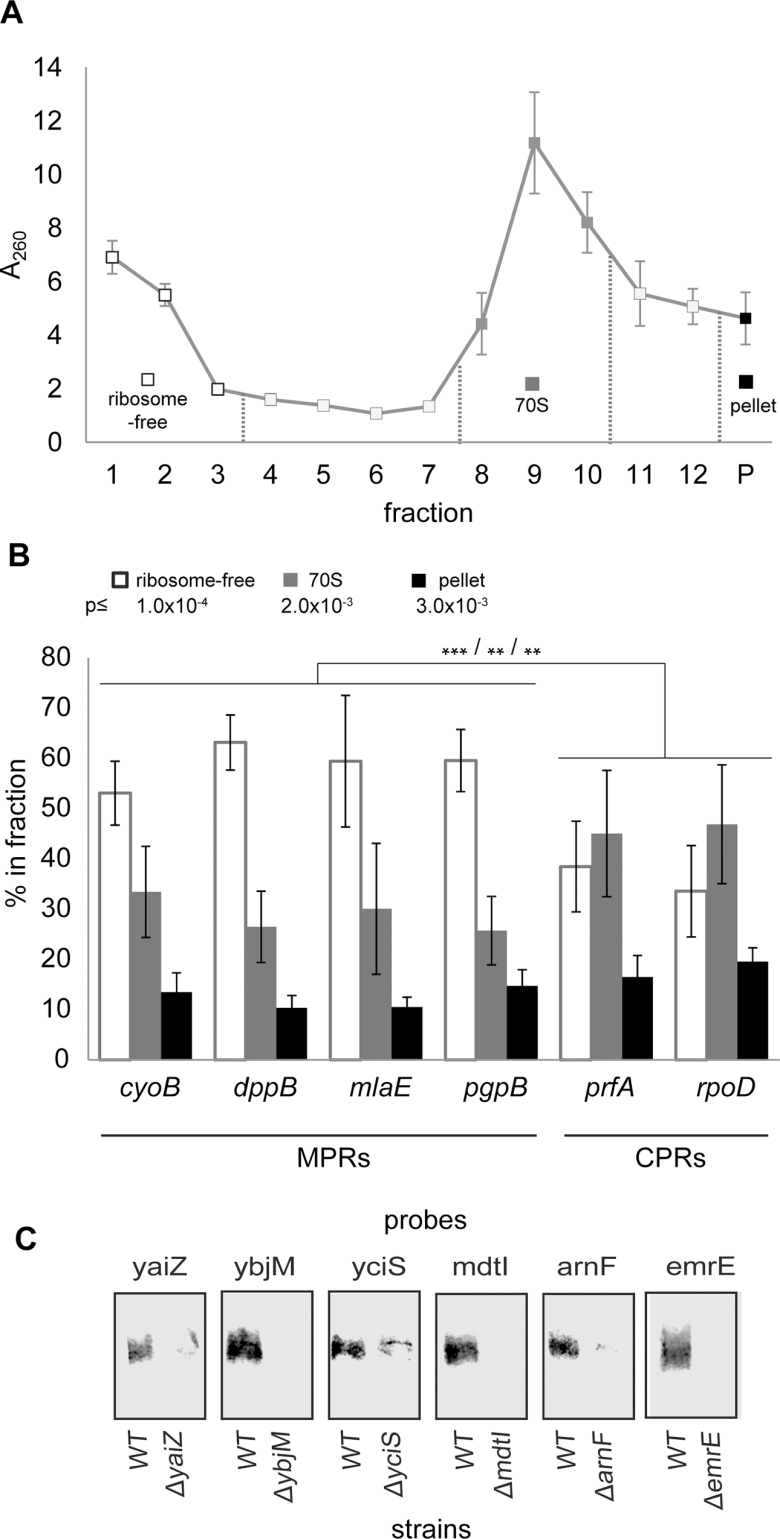
Small-scale analysis of mRNA distribution in fractionated *E*. *coli*. Cell extract was fractionated through a 7.5–25% sucrose gradient. **(A)** A_260_ of the sucrose gradient fractions (triplicates, error bars indicate SD). **(B)** RNA was prepared from the total cell extract, the pooled ribosome-free fractions (1–3), the 70S fractions (8–10), and from the pellet. The relative concentration of each of the indicated transcripts was calculated as 2^extract Ct^ / 2^extract^. The portion of each transcripts in each fraction was calculated as: [transcript]_fraction_*RNA(μg)_fraction_ / [transcript]_extract_*RNA(μg)_extract_. Error bars indicate SD, n = 3. Student t-test p-values for differential distribution of MPRs and CPRs to each of the fractions are depicted along with the color key. **(C)** Northern analysis of randomly selected MPRs in the ribosome-free fraction (1–3). This experiment was conducted twice and the results shown are representative.

**Fig 2:**
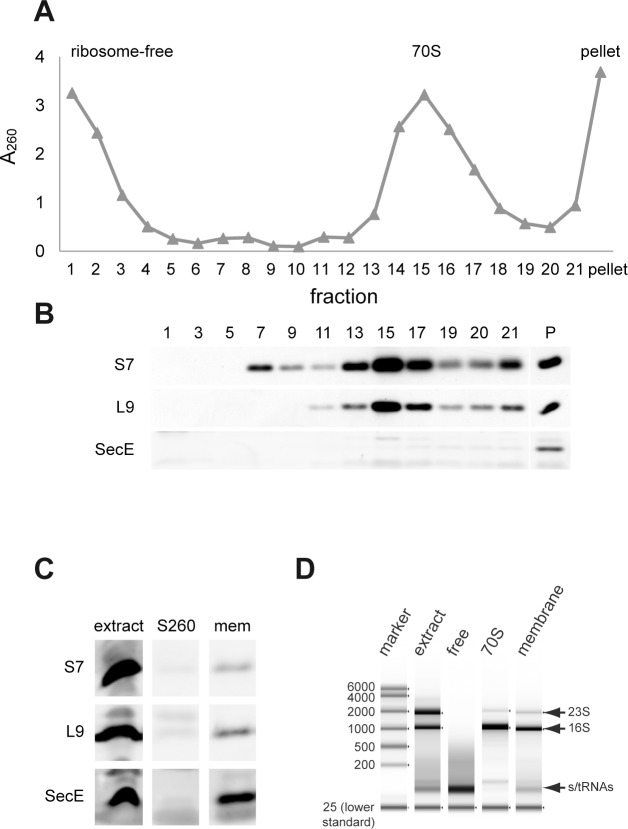
Large scale fractionation of *E*. *coli*. Cells were disrupted in a buffer containing 15 mM Mg^+2^ and cell extract was fractionated by either 7–22% sucrose gradient centrifugation or by floatation through high-density sucrose solution. **(A)** A_260_ of the sucrose gradient fractions in the presence of 15 mM Mg^2+^. **(B)** Analysis of the sucrose gradient fractions by Western blotting with antibodies against S7 (ribosomal small subunit protein), L9 (ribosomal large subunit protein) or SecE (integral membrane protein). **(C)** Analysis of the cell extract, the ultracentrifugation supernatant (S260) and the floatation-purified membranes (from the pellet p260) by Western blotting as described in **(B)**. **(D)** Tapestation analysis of RNA extracted from total cell extract (extract), from the sucrose density gradient fractions: ribosome-free (free) and 70S-associated (70S) RNA and from the floatation-purified membrane (membrane).

In this work, we study the subcellular distribution of mRNAs in *E*. *coli* by cell fractionation for the first time. Initially, we examined if this approach is feasible in a small scale, by characterizing the mRNA content in *E*. *coli* extracts and their subcellular fractions. We utilized a small volume sucrose density gradient (Fig 1A) and examined the relative amount of several randomly selected mRNAs by qPCR. Fig 1B shows that of the analyzed set of MPRs and CPRs, the formers were specifically and substantially enriched in the ribosome-free fractions, while the CPRs were slightly overrepresented in the 70S ribosomal fraction. If this trend is generally true, it may imply that there is a pool of MPRs in the cytosol. The crude pellet fraction might also contain heavy complexes and some very heavy cytosolic polysomes, in addition to the membrane. Therefore, it would be expected that the pellet fraction is slightly enriched by CPRs if CPRs are preferentially translated by large cytosolic polysomes. Therefore, the pellet fraction does not represent only the membranes and in the following studies, we analyzed membranes that were purified by floatation of the P260 pellet that contains all ribosomes and membranes (see Fig 2C). The qPCR analysis of the distribution of randomly selected transcripts shows that at a steady state, 10–20% of the total mRNAs migrated to the gradient pellet, 30–45% migrated with 70S ribosomes, 30–60% migrated to the ribosome-free fraction, and thus, only a minority of mRNA seems to exist in other fractions that were not analyzed (Fig 1A, fractions 4–7 and 11–12). Further analysis of the polysomal fractions in independent experiments showed that the distribution of mRNAs to these fractions is similar to their distribution to the 70S ribosome fractions (S1 Fig). Clearly, these results indicate that under our fractionation conditions the relative amount of mRNA in the various fractions that we chose to analyze further (see later) is significant and representative. Fig 1C demonstrates by Northern blotting with probes to several relatively short mRNAs that they indeed tolerated, at least to some extent, the fractionation procedure.

Intrigued by the MPR-distribution results (Fig 1B) we performed a large-scale experiment. Wild-type *E*. *coli* cells were disrupted and fractionated in a preparative manner by sucrose gradient centrifugation (Fig 2A and 2B). In addition, membranes were purified by floatation of the P260 pellet obtained by ultracentrifugation of the cell extract (Fig 2C). The fractionations were conducted in the presence of relatively high Mg^2+^ concentrations, because at low Mg^2+^ concentrations the ribosomal subunits dissociate and the interaction of mRNAs with ribosomes is disrupted [16]. In the sucrose gradient, the ribosomes migrated mainly in fractions 13–18 (Fig 2A and 2B, upper panels), whereas the upper part of the gradient (ribosome-free fractions 1–5) is likely enriched with tRNAs and sRNAs. Membranes are efficiently concentrated in the pellet at the bottom of the gradient as confirmed by Western blot with anti-SecE antibodies (Fig 2B, lower panel). However, as explained above, this pellet also contains other high molecular weight particles, such as large polysomes. Therefore, we used the flotation procedure as a source of purified membranes. The indicated fractions in Fig 2C were evaluated by several markers. The floated membranes contain the IMP SecE (Fig 2C, lower panel), as expected, and also ribosomes, as shown by Western blotting with antibodies to proteins of the 2 ribosomal subunits (Fig 2C, upper panels). Regarding the membrane-associated ribosomes, it is currently unknown whether all of them are associated with the membrane in a specific manner, since they may also interact nonspecifically with the membrane or membrane proteins during the preparation of cell extracts or through their 23S RNA via the SecYEG complex [17]. Similarly, cytosolic ribosomes (Fig 2A, fractions 13–18) that were isolated by sucrose gradient centrifugation may include ribosomes that were detached from the membrane during the fractionation process. Therefore, it is likely that the results with purified membranes and cytosolic ribosomes might yield somewhat noisy data. Nevertheless, as will be shown, these concerns did not preclude reasonable analyses of the mRNA-distribution patterns, especially in the ribosome-free fractions (Fig 2A, fractions 1–5). In addition to the total extract and the purified membranes, two density gradient regions were collected: one containing the soluble ribosome-free fraction (Fig 2A, fractions 1–5) and the other containing pooled cytosolic ribosomal fractions (Fig 2A, fractions 13–18). RNA was prepared from the pooled fractions and analyzed by Tapestation (Fig 2D). For initial evaluation of its content, we sampled the ribosome-free soluble fraction and analyzed the reverse transcribed RNA by PCR with primers flanking several ORFs. The results suggest that this fraction contains intact transcripts (S2 Fig), as also implied from the small-scale Northern blot analysis (Fig 1C). Previous studies demonstrated that partially cleaved transcripts are degraded almost instantaneously by exoribonucleases [18, 19], suggesting that the identified mRNAs are largely intact.

Next, the RNAs prepared from all the fractions and the total extract were subjected to NGS. The sequencing data were grouped into MPRs and CPRs (see Methods and S5 Table) and here we describe their subcellular distribution as the ratio of their amounts in the indicated fraction from their respective amounts in the total extract. For each experimental set, we followed the distribution of an identical group of mRNAs, thus enabling us to faithfully compare the distribution patterns of the same mRNAs in the different fractions (S6 Table). For the analyses of mRNA distribution in wild-type *E*. *coli*, we followed 201 MPRs and 766 CPRs, which were detected in all the fractions. In addition, we analyzed the full, non-overlapping data sets (S5 Table) for the ribosome-free soluble fraction and for the membrane, and the results were consistent with those obtained from analyzing the overlapping data sets. All the RNA-seq results are described in the following sections.

### Membrane distribution of MPRs and CPRs

**Fig 3:**
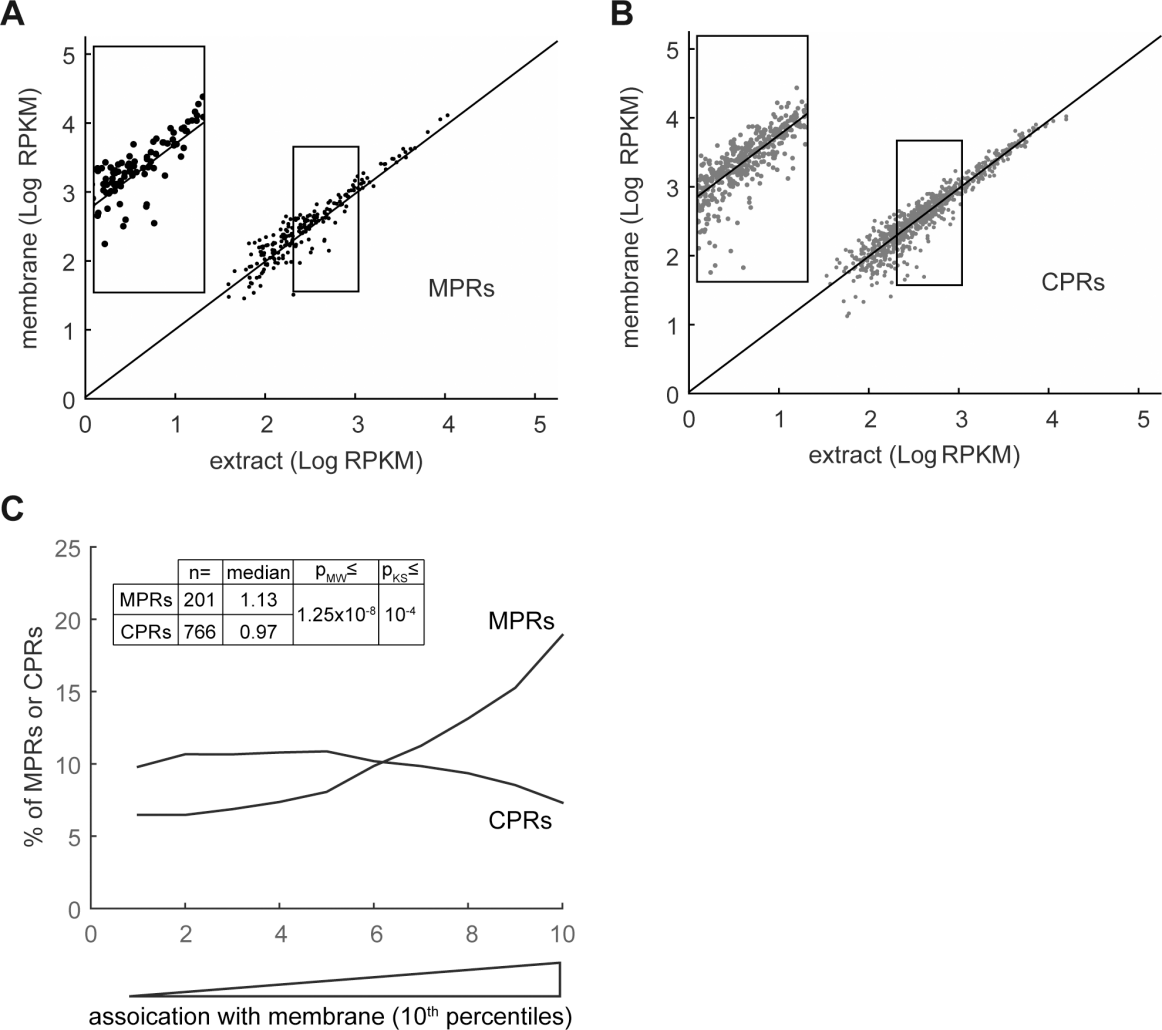
Distribution of MPRs and CPRs to the membrane fraction. RNA was extracted from the total extracts and from the purified membranes and analyzed by RNA-seq. **(A)** and **(B)** Reads per kilobase per million mapped reads (RPKMs) in the membrane fraction were plotted as a function of the total extract RPKMs for MPRs and CPRs, respectively. A linear regression plot of all identified mRNAs is presented as reference. Insets show magnification of the framed regions. **(C)** The enrichment of mRNAs on the membranes was calculated as [RPKM_membrane_ / RPKM_extract_]. The quota of MPRs and CPRs in each 10^th^ percentile along the experimental landscapes is presented as a moving average plot. Median values, and Mann-Whitney (MW) and Kolmogorov-Smirnov (KS) p-values for different membrane enrichment means and distributions of MPRs and CPRs are presented.

Most IMPs are translated on the membrane, by membrane-associated ribosomes [4], representing an evolutionarily conserved process [20, 21]. Therefore, it is reasonable to assume that MPRs are overrepresented on the membrane compared with CPRs, which can be translated by cytosolic ribosomes. As shown here, despite the concern raised above regarding the fractionation quality, MPRs were found, on average, overrepresented in the membrane fraction (Fig 3A) compared with CPRs (Fig 3B). This is exemplified in Fig 3C. Of 201 MPRs, 92 were within the 30% most membrane-enriched RNAs (p-value = 4.5x10^-8^, or 6.1x10^-5^ after FDR correction, see Experimental Procedures for details). In contrast, only 39 MPRs were among the 30% least membrane-associated RNAs. Analysis of the entire membrane data set revealed similar results (S3 Fig). As noted above, this is not surprising since IMPs are known to be inserted into the membrane co-translationally [22]. Notably, we also found membrane-associated CPRs (Fig 3B) but to a lesser extent than MPRs (Fig 3C).

### Distribution of CPRs and MPRs to the fraction of cytosolic ribosomes

**Fig 4:**
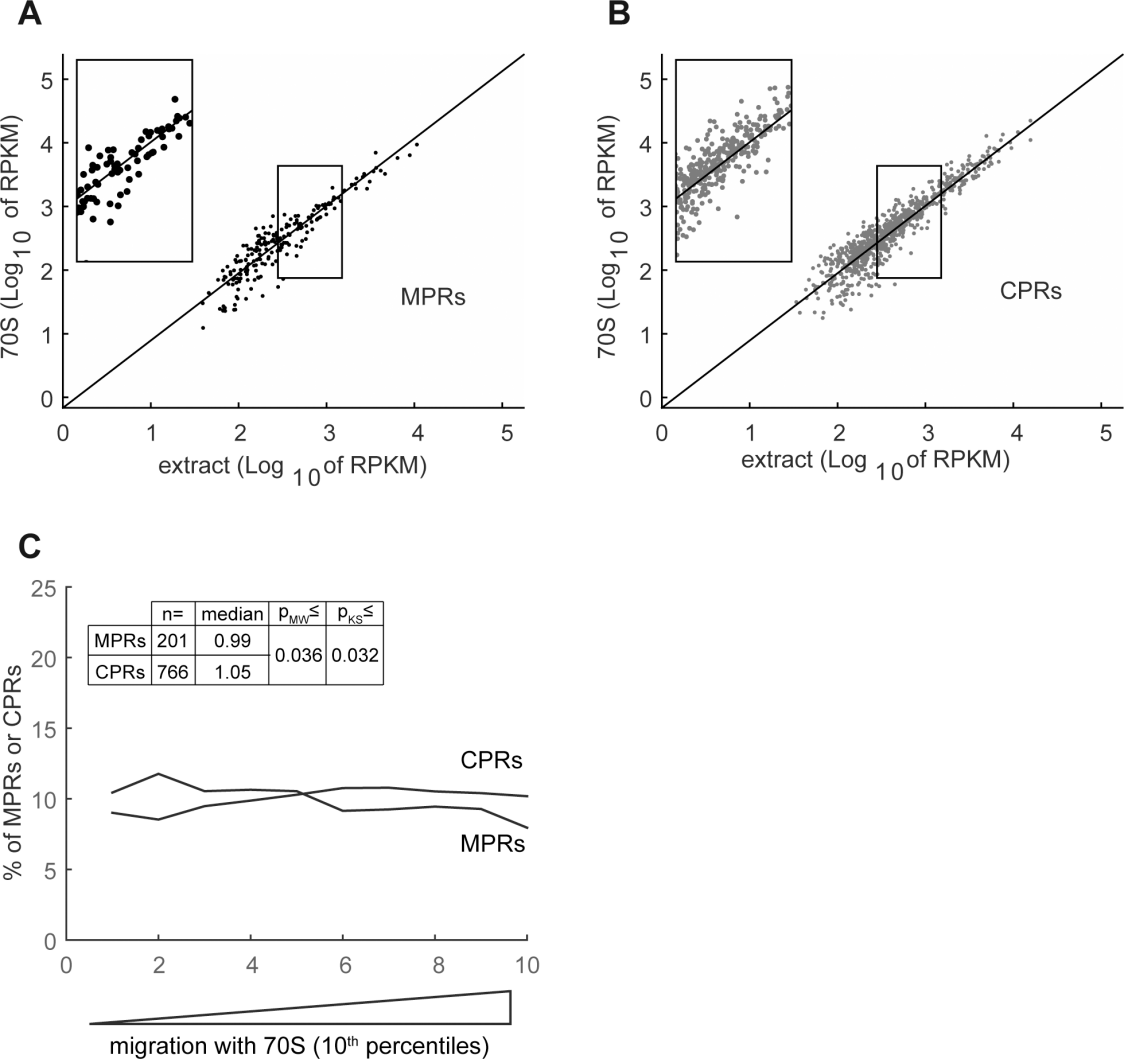
Distribution of MPRs and CPRs to the fraction of cytosolic ribosomes. *E*. *coli* extract was fractionated by 7–22% sucrose gradient centrifugation. Cytosolic ribosomes-containing fractions (70S) were pooled, RNA was extracted from total cell extract and from the pooled fractions and analyzed by RNA-seq. **(A)** and **(B)** Reads per kilobase per million mapped reads (RPKMs) in the 70S fraction were plotted as a function of extract RPKMs for MPRs and CPRs respectively. A linear regression plot of all identified mRNAs is presented as reference. Insets show magnification of the framed regions **(C)** The enrichment of all the mRNAs in the 70S fraction was calculated as [RPKM_fraction_ / RPKM_extract_]. The quota of MPRs and CPRs in each 10^th^ percentile along the experimental landscapes is presented as a moving average plot Median values, and Mann-Whitney (MW) and Kolmogorov-Smirnov (KS) p-values for different 70S enrichment means and distributions of MPRs and CPRs are presented.

Next, we sequenced and analyzed the relative amounts of MPRs and CPRs in the cytosolic ribosomal fraction. Here we collected and prepared RNA for sequencing from the main ribosome-containing fractions of the sucrose density gradient according to their absorbance at 260 nm (Fig 2A, fractions 13–18). Clearly, ribosomal RNAs represent most of the RNA content in this fraction (data not shown). Nevertheless, we succeeded to faithfully analyze the distribution of a significant number of mRNAs. Fig 4A–4C show almost no difference between the relative distribution of CPRs and MPRs to the ribosomal fraction.

### CPRs and MPRs in the ribosome-free cytosolic fraction

**Fig 5:**
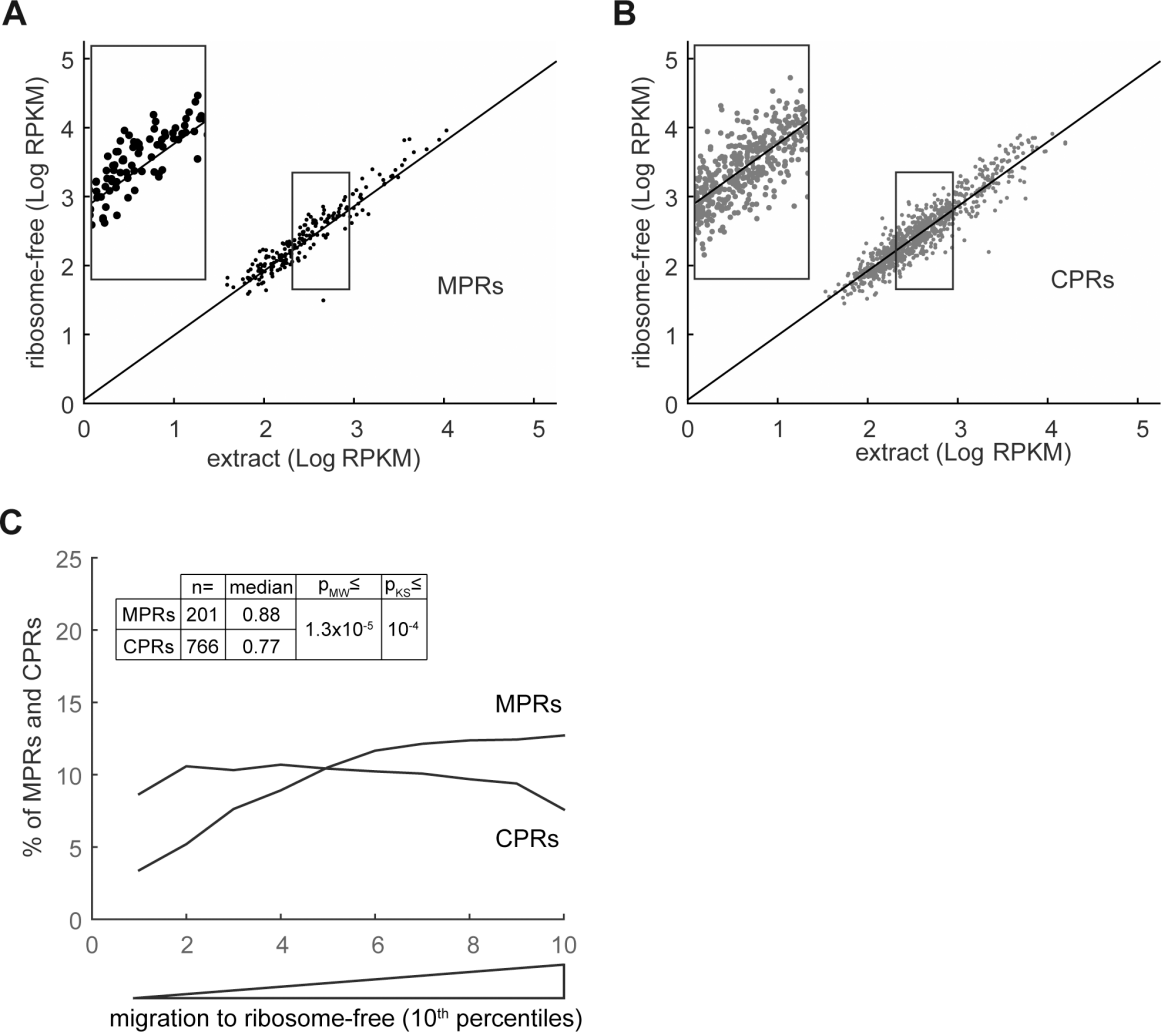
Distribution of MPRs and CPRs to the ribosome-free fraction of the sucrose gradient. *E*. *coli* total extracts were loaded on a 7–22% sucrose gradient, ultracentrifuged, and fractions 1–5 from the top (ribosome-free) were pooled. RNA was prepared from the total cell extract and from the pooled ribosome-free fractions and analyzed by RNA-seq. **(A) and (B)** RPKMs in the ribosome-free fraction were plotted as a function of extract RPKMs for MPRs and CPRs respectively. A linear regression plot of all identified mRNAs is presented as reference. Insets show magnification of the framed regions **(C)** The enrichment of mRNAs in the ribosome-free fractions was calculated as [RPKM_fraction_ / RPKM_extract_]. The quota of MPRs and CPRs in each 10^th^ percentile along the experimental landscapes is presented as a moving average plot. Median values, and Mann-Whitney (MW) and Kolmogorov-Smirnov (KS) p-values for different cytosolic, ribosome-free,enrichment means and distributions of MPRs and CPRs are presented.

As mentioned earlier, it is likely that the ribosome-free soluble fractions (Fig 2A, fractions 1–3) of the density gradient contain mainly tRNAs and ncRNAs. Nevertheless, inspired by our qPCR results (Fig 1B), we decided to sequence and analyze the content by RNA-seq. Generally, the results revealed that the ribosome-free fraction contains a significant amount of mRNA. Owing to the very low ribosomal RNA content in the top fractions of the gradient, mRNAs were sequenced much more efficiently compared to the sequencing of fractions that contained ribosomes (e.g. cytosolic ribosomal fraction and membranes). Initially, we analyzed the shared (limited) data set (S6 Table) and the results resembled our qPCR studies (Fig 1B), as they showed that the ribosome-free fractions are enriched with MPRs (compare Fig 5A with 5B). As summarized in Fig 5C, 78 of the 201 MPRs were within the 30% most enriched transcripts in the ribosome-free fractions (for mRNAs above the 5^th^ decile the p-value = 7.6x10^-8^, or 1.2x10^-4^ after FDR correction). Only 35 MPRs were among the 30% least enriched transcripts in the ribosome-free fraction. Analysis of the entire data set in this fraction (S5 Table) revealed a similar enrichment of MPRs (S4 Fig). These results are surprising because they suggest that on average, many MPRs avoid interaction with cytosolic ribosomes.

Together, the mRNA distribution results suggest that there are 2 major pools of MPRs in fractionated cells, in the cytoplasmic ribosome-free fraction and on the membrane. Accordingly, we hypothesize that MPRs are delivered from the cytoplasm to the membrane in a translation-independent manner. The machinery and underlying mechanism of such a pathway are unknown. In this regard however, the SRP-system has been proposed to play a crucial role in the translation-dependent targeting of MPRs, and we asked whether this system is also involved either directly or indirectly, in mediating MPR targeting to the membrane in our experimental setup.

### Effects of depletion of FtsY or kasugamycin (Kas) treatment on the membrane distribution of mRNAs

**Fig 6:**
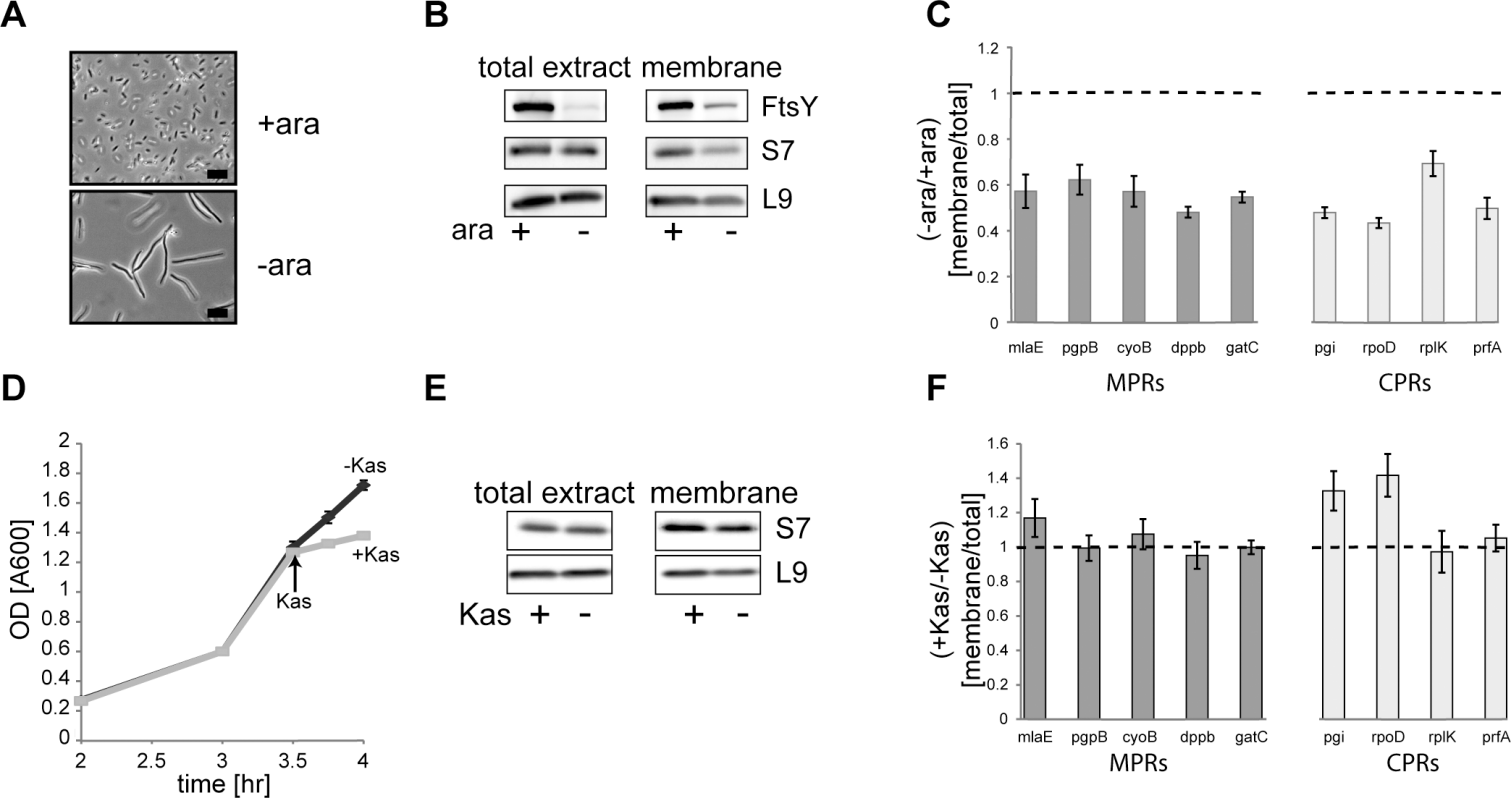
Membrane distribution of MPRs and CPRs in cells depleted of FtsY or treated with Kas. **(A)** Light microscopy of the FtsY-depletion *E*. *coli* IY28, grown with or without ara, **(B)** FtsY depleted (-ara) and non-depleted (+ara) cells were disrupted and membranes were purified by flotation. The total extracts and the membrane fractions were analyzed by Western blotting with antibodies against the indicated proteins. Equal amount of protein (30 μg) were loaded in all lanes. **(C)** qPCR analysis of the amount of several mRNAs in the purified membranes is presented as the ratio of enrichment in the membrane versus the total extract (-/+FtsY). **(D)** Growth curves before (3.5 h) and after addition of Kasugamycin (Kas) (indicated by an arrow) for 30 min. **(E)** Kas treated cells were disrupted and membranes were purified by flotation. The total extract and membrabne fractions were analyzed by Western blotting with antibodies against the indicated proteins. Equal amount of protein (30 μg) were loaded in all lanes. **(F)** qPCR analysis of the amount of several mRNAs in the purified membranes is presented as ratio of enrichment in the membrane versus the total extract (+/-Kas). Error bars indicate SEM (n = 3–5).

Previously, we showed that the SRP receptor, FtsY is required for translation of membrane proteins [23, 24] and targeting of ribosomes to the membrane [25, 26]. We reasoned that utilizing the FtsY-depletion phenotype may reveal how MPRs targeting is affected under conditions of specifically impaired membrane association of ribosomes and membrane protein translation. For this we utilized the FtsY-depletion strain IY28 [27] grown with or without the FtsY inducer arabinose (Fig 6A). We chose relatively mild FtsY-depletion conditions and the depletion was confirmed by Western blotting of samples from total extracts and purified membranes (Fig 6B). The results also show that, as expected, the amount of membrane associated ribosomes (ribosomal proteins S7 and L9) was reduced in FtsY-depleted membranes (Fig 6B, right panel). Next, RNA was prepared from the total extracts and from the flotation-purified membranes and analyzed by qPCR using primers complementary to several MPRs and CPRs. Fig 6C shows that FtsY-depletion generally reduced the amount of all mRNAs on the membrane, including both CPRs and MPRs. This experiment thus shows that the changes in the quantities of mRNAs on the membrane and in the amounts of membrane-associated ribosomes correlate, raising a possibility that at least a portion of the identified membrane-associated mRNAs is ribosome bound. However, other tentative explanations for the overall reduced amount of membrane-associated mRNAs under these conditions cannot be excluded, such as the possibility that the decrease in the amount of membrane associated mRNAs is a stress phenomenon, which is caused by depletion of any SRP-system component [28, 29].

Therefore, we examined the effect of the antibiotic Kas, which is also known to confer stress [30]. Kas is an aminoglycoside antibiotic that inhibits protein synthesis during the step of translation initiation [31]. Interestingly, Kas inhibits translation of canonical transcripts containing a 5'-UTR with a Shine Dalgarno (SD) motif, but probably not of leaderless transcripts. However, the exact mechanism that underlies its translation inhibition activity is still unknown [32]. Fig 6D shows that Kas addition had an instantaneous inhibitory effect on growth with no effect on the amount of membrane-associated ribosomes (Fig 6E, right panel). However, in contrast to the results with cells depleted of FtsY, Kas treatment had almost no effect on the distribution of the various mRNAs to the membrane fraction (Fig 6F). These results suggest that the steady state amount of mRNA on the *E*. *coli* membrane is not affected by a relatively short Kas treatment and translation initiation arrest. This lends support to the hypothesized translation-independent mRNA localization. The results of these experiments showed no preference for MPRs or CPRs in how they were influenced by FtsY-depletion, suggesting that the effect is indirect, probably through the role of FtsY in membrane protein biogenesis. While searching for factors that may have a direct role in the biogenesis of MPRs specifically, we have recently discovered that cold shock proteins (CSPs) such as CspE specifically interact with MPRs *in vitro* and *in vivo* [15]. Therefore, the role of CspE in the subcellular distribution of MPRs was examined next.

### Subcellular distribution of CspE

**Fig 7:**
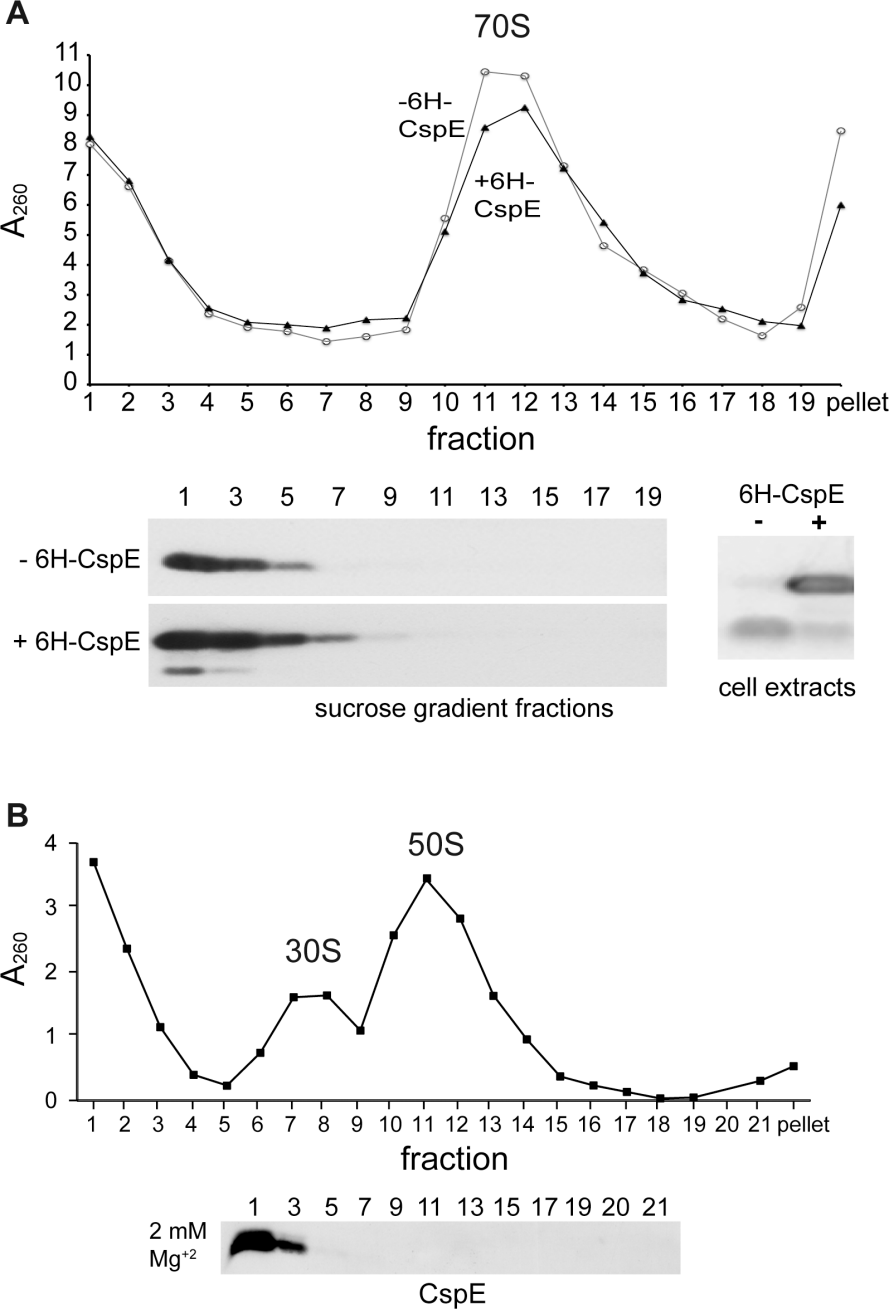
Subcellular distribution of CspE and 6His-CspE. **(A)** Cell extracts of wild type *E*. *coli* with or without plasmid expressing 6His-CspE (3.3 mg of total protein) were fractionated by a large scale 7.5%-25% sucrose gradient in buffer containing 15 mM Mg^2+^. Upper panel, A_260_ of the sucrose gradient fractions is shown. Lower panel, analysis of the sucrose gradient fractions (left) and the cell extracts (right) by Western blotting with anti-CspE antibodies. 3 times more of the sucrose gradient fraction samples were loaded in the case of wild type compared to those of cells over-expressing 6His-CspE. **(B)** Upper panel. Wild type cells were disrupted and fractionated by 7–22% sucrose density gradient centrifugation in the presence of only 2 mM Mg^+2^. A_260_ of the sucrose gradient fractions is shown. Lower panel, analysis of the sucrose gradient fractions by Western blotting with anti-CspE antibodies.

As a first attempt to investigate whether CSPs are involved in the biogenesis of MPRs and especially in their distribution to the ribosome-free fraction, we analyzed sucrose gradients for the subcellular distribution of endogenous CspE and of plasmid encoded 6His-CspE (Fig 7). The results indicate unambiguously that both the native CspE and the plasmid born 6His-CspE were almost completely absent in the ribosomal fractions (Fig 7A, low panel). Instead, both forms of CspE were found in ribosome-free fractions at the top of the gradient, which were also found enriched with MPRs. This experiment was then repeated at low Mg^2+^ concentrations that promote dissociation of ribosomes to their small (30S) and large (50S) subunits, and release of mRNAs (Fig 7B). The results with CspE were similar to those obtained under high Mg^2+^ conditions. CspE migrated mainly in the top, ribosome-free fractions (Fig 7B, lower panel), suggesting no specific association with separate ribosomal subunits. Taken together, these results, combined with our previous studies, raise the possibility that the observed interaction of MPRs with CSPs [15] likely occurs in the ribosome-free fraction and this notion was tested next.

### Association of MPRs with CspE-6His in the ribosome-free fraction

**Fig 8:**
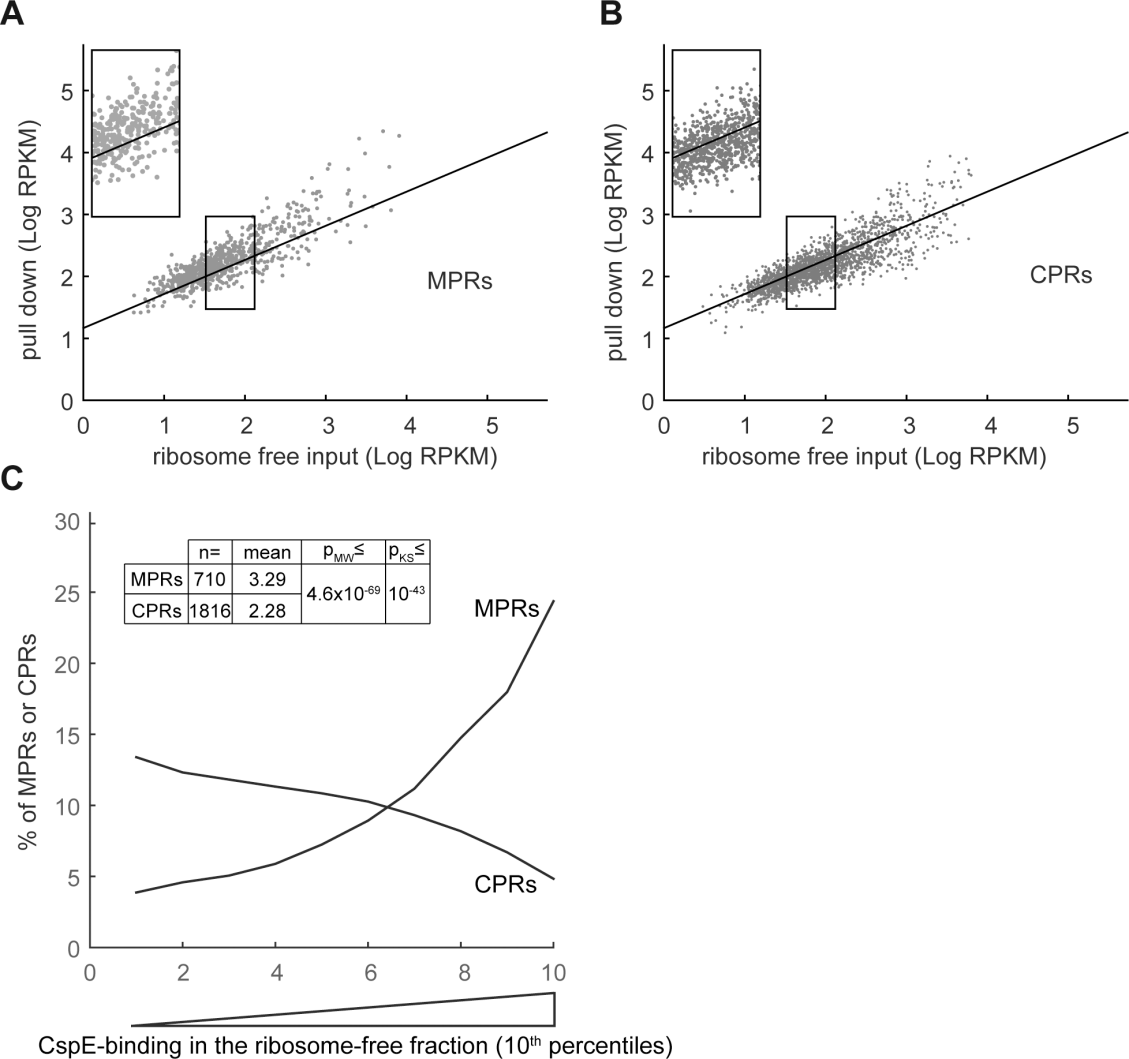
CspE-6His-associated mRNAs in the ribosome-free fraction. Wild type *E*. *coli* expressing CspE-6His were disrupted in the presence of high [Mg^2+^], and the cell extract was fractionated by sucrose gradient centrifugation. The top, ribosome-free fractions (1–5) were pooled and RNA-CspE-6His complexes were purified by TALON beads. RNA was prepared from the pooled fractions before the pull-down (input) and after the imidazole-elution and subjected to RNA-seq. A total of 3408 mRNAs were analyzed, including 605 MPRs and 1613 CPRs. **(A)** and **(B)** RPKMs in the CspE-bound fraction were plotted as a function of input RPKMs for MPRs and CPRs respectively. A linear regression plot of all identified mRNAs is presented as reference. Insets show magnification of the framed regions **(C)** The enrichment of mRNAs in the CspE-bound fraction was calculated as [RPKM_CspE-bound_ / RPKM_input_]. The quota of MPRs and in each 10^th^ percentile along the experimental landscapes is presented as a moving average plot. Mann-Whitney (MW) and Kolmogorov-Smirnov (KS) p-values for different CspE-binding means and distributions of MPRs and CPRs are presented.

Previously, we have shown by qPCR that CspE-6His co-purified with several MPRs from *E*. *coli* total extracts and we also confirmed the results by RNA-seq of the entire pool of 6His-CspE-bound mRNAs [15]. Since CspE (and also CspE-6His) migrates in the ribosome-free soluble fraction of the sucrose density gradient (Fig 7), as do MPRs (Fig 5), it is expected that the previously observed MPR-CspE association occurs in that fraction. To assess this notion, we fractionated cells expressing CspE-6His and performed metal affinity pull-down with the pooled ribosome-free fractions. The pulled-down RNA was sequenced and 3408 mRNAs were detected, including 1613 CPRs and 605 MPRs. The results indicate that, compared to their amount in the input sample, MPRs were highly enriched in the CspE-6His pull-down material (compare Fig 8A with 8B). According to the analysis of the CspE-6His best binders (Fig 8C), CspE unequivocally binds MPRs preferentially in the ribosome free soluble fraction (p-value = 2.2x10^-30^ or 2.9x10^-27^ after FDR correction). Analysis of the CspE-6His weak binders shows that CspE is a significant non-binder of CPRs (p-value = 2.2x10^-28^ or 2x10^-26^ after bonferroni correction). In summary, the pull-down results showed that, on average, MPRs interact with CspE markedly better than CPRs in the ribosome-free fraction, as shown previously with total extracts [15]. The question whether CspE, through its specific interaction with MPRs affects their subcellular distribution was addressed next.

### Effect of CSPs on the subcellular distribution of MPRs and CPRs

**Fig 9:**
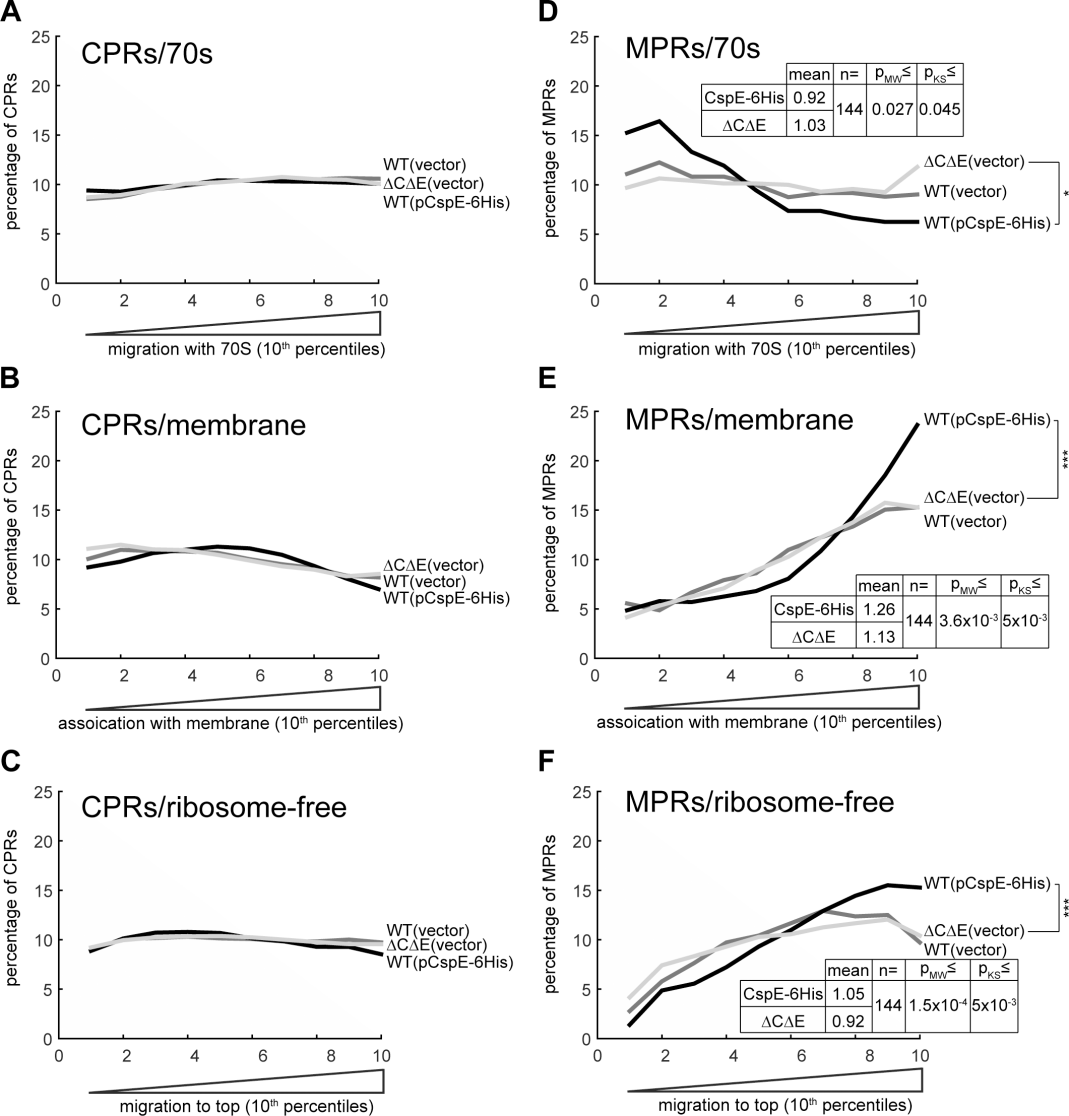
Effects of CspE-6His over-expression or *cspC/cspE* double deletion on the distribution of CPRs and MPRs. Wild type (WT) *E*. *coli* harboring an empty vector (dark grey) or a CspE-6His expressing plasmid (black) and the double deletion mutant *E*. *coli* Δ*cspC/*Δ*cspE* harboring an empty vector (light grey), were disrupted and fractionated either by sucrose gradient, for isolation of ribosome-free cytosolic and ribosome-bound RNAs, or by membrane floatation for purification of membrane-associated RNA. RNA was extracted from the total cell extract and fractions of each strain and analyzed by RNA-seq. 976 mRNAs were analyzed in all strains and fractions, including 586 CPRs **(A—C)** and 144 MPRs **(D—F)**. The enrichment of all the detected mRNAs in each fraction was calculated as [RPKM_fraction_ / RPKM_extract_]. The quota of MPRs and CPRs in each 10^th^ percentile along the experimental landscapes is presented as a moving average plot for migration with cytosolic ribosomes **(A)** and **(D)**, association with the membrane **(B)** and **(E)** and migration in the ribosome-free fraction **(C)** and **(F)**. Mann-Whitney (MW) and Kolmogorov-Smirnov (KS) p-values for different means and distribution of MPRs in fractions (CspE-6His expression vs. *cspEcspC* deletion) are presented.

*E*. *coli* has 9 CSP homologs (CspA-CspI) [33] and it is noteworthy that several of them belong to a common regulatory network (see also S6 Fig). Thus, studying their individual role(s) *in vivo* is challenging. Of the relatively highly expressed CSPs, our recent studies demonstrated that CspC and CspE are U-rich RNA binders, whereas CspA exhibits only marginal binding [15]. Therefore, we investigated whether CspC or CspE, or both play a role in the biogenesis of MPRs, by utilizing our previously constructed *E*. *coli* Δ*cspE*/Δ*cspC* and plasmids for expression of CspE-6His [15]. Specifically, we isolated membrane, ribosomal, and ribosome-free soluble fractions from wild-type *E*. *coli* harboring an empty vector or a plasmid encoding CspE-6His or from *E*. *coli*Δ*cspE/*Δ*cspC*. RNA prepared from all the fractions was sequenced. Initially, we used clustering analysis to examine whether CspE overexpression or *cspE*/*cspC* deletion affected the steady state amounts of the sequenced mRNAs and how these effects correlate with the strengths of CspE-6His binding. The results show clearly that although the amount of many mRNAs varies between the strains, the differences and the strengths of CspE-6His binding do not correlate (S5 Fig). Next, we analyzed the effects of CspE-6His overexpression and *cspE/cspC* deletion on the subcellular distribution of mRNAs (Fig 9). In this set of experiments, we identified and analyzed an overlapping group (same mRNAs that were detected in all the fractions of all the 3 strains) of 976 mRNAs, including 586 CPRs and 144 MPRs (S7 Table). The overall analysis clearly revealed that the subcellular distribution patterns of CPRs remained unaffected by deletion or overexpression of CSPs (Fig 9A–9C). Remarkably, in contrast, the expression of CspE had a marked effect on the subcellular distribution of MPRs. In the cytosolic ribosomal fraction, CspE-6His expression further reduced the amount of MPRs, whereas on the membranes and in the ribosome-free fraction, CspE-6His expression increased the amount of MPRs (Fig 9D–9F). Finally, there were no appreciable effects on the distribution in a strain harboring the double deletion of *cspE* and *cspC*, but this may not be surprising, since in this strain the expression of other CSPs is induced (S6 Fig).

**Fig 10:**
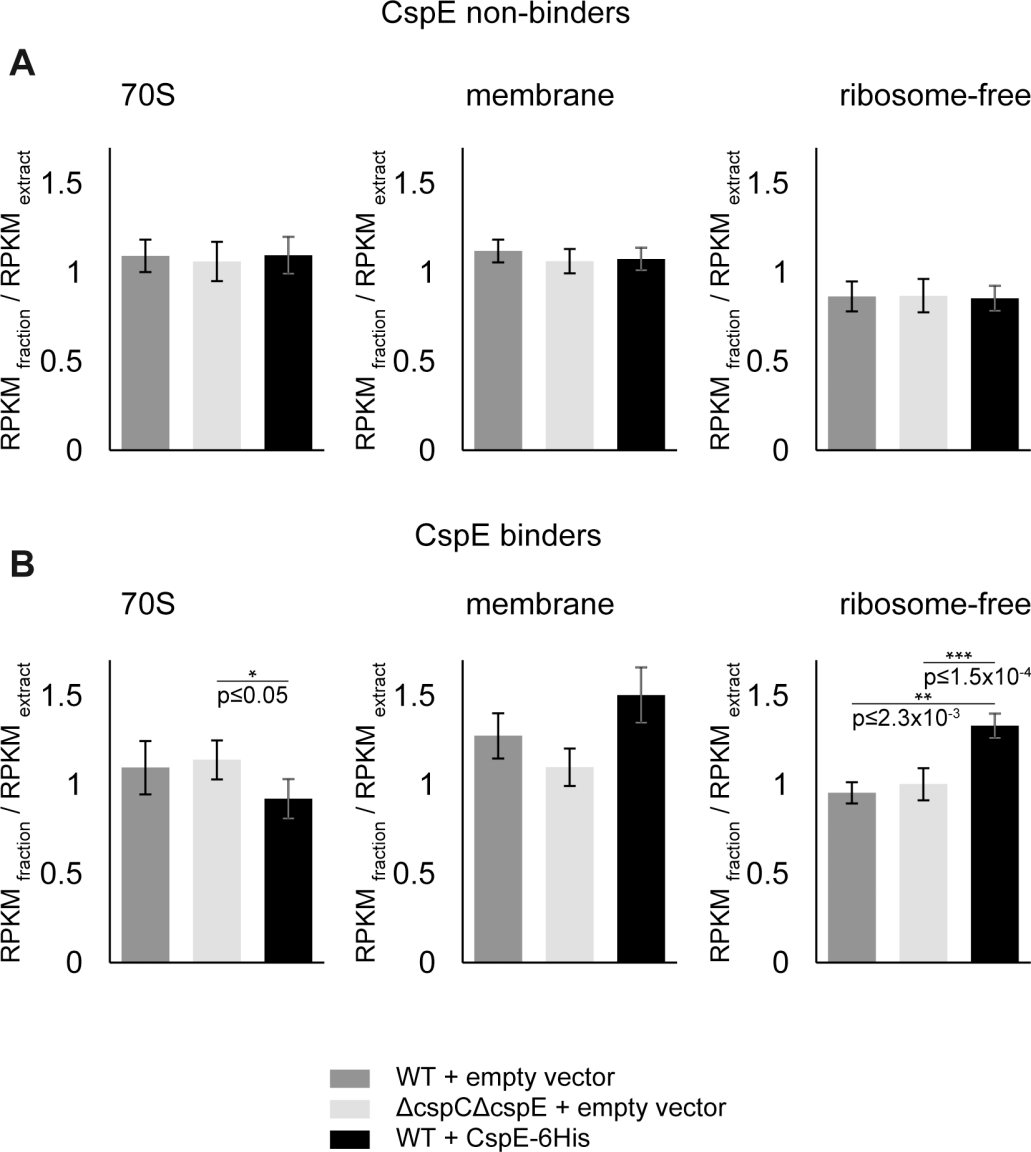
Correlation between CspE-6His association of MPRs and their subcellular distribution. Wild type (WT) *E*. *coli* harboring an empty vector (dark grey) or a CspE-6His expressing plasmid (black) and *E*. *coli* Δ*cspC/*Δ*cspE* harboring an empty vector (light grey), were disrupted and fractionated as described above (**Fig 9**). The average enrichment [RPKM_fraction_ / RPKM_extract_] of CspE-6His non-binding MPRs (19 mRNAs), and of CspE-6His binding MPRs (17 mRNAs) in each fraction is presented (error bars indicate SEM). Mann Whitney test was used to calculate p values.

These results also show that the subcellular distribution of certain MPRs is considerably affected by CspE-6His overexpression, whereas other MPRs remain unaffected. If the effect of CspE-6His on the distribution is direct, through physical interaction between the MPRs and the protein, then the strength of binding and the extent of the effect on the distribution may correlate. To investigate the reason for this heterogeneity, we selected two groups of MPRs: strong binders and weak binders. Weak CspE-binders are mRNAs that were identified as 30% least-bound to CspE. Since most MPRs bind CspE, as expected, a low number of only 31 MPRs were found to be weak CspE binders, and accordingly, we selected also an equal-sized group of mRNAs that were found in the top of the list of CspE-bound mRNAs. Then, we further selected from these mRNAs the ones that were detected in all the fractions of all the 3 strains (wild type *E*. *coli*, CspE-6H over-expressing cells and *E*. *coli* Δ*cspE*/Δ*cspC*). This led to an overlapping data set that includes 19 CspE weak binders and 17 CspE strong binders. We then analyzed the extent of influence of CspE deletion and overexpression on their subcellular distribution. Fig 10 shows that on average, only the distribution of the strong CspE binders was affected by CspE-6His expression (the effect on the distribution to the membrane was just below statistical significance), suggesting a direct involvement of CspE. Notably however, despite the small size of the examined groups, the results show clearly that the distribution of the strong binders to the ribosome-free fraction was significantly affected by CspE overexpression (p-value = 0.001, single factor ANOVA).

Next, we asked whether the CspE binding-strength reflects the number of recognition sites for cold shock proteins within the various MPRs. The specificity of the interaction between CSPs and ssDNA or RNA has been extensively studied [34]. Based on some of those studies that appear to be more relevant to CspE, we analyzed the number of recognition elements in our defined MPR groups of strong and weak CspE binders. The results clearly show that the strong binders harbor significantly more CSP-recognition stretches, enriched in T/U, than those that bind CspE poorly (Table 1 and S8 Table).

**Table 1:** Number of CSP-recognition elements in our defined MPR groups of strong and weak CspE binders (see S8 Table for the entire data).

Species	Protein	Recognized sequence	Ref.	Weak binders *	Strong binders **
*B*. *subtilis*	CspB	TTCTTTT	[52]	0	2 (3)
*B*. *subtilis*	CspB	GTCTTTT/G	[53]	2 (2)	5 (6)
*B*. *subtilis*	CspB	GUCUUUU/A or UUUUUU	[54]	3 (3)	8 (9)
*T*. *maritima*	*Tm*Csp	A 7-base long sequence of Ts or Us interrupted by a single purine	[55]	3 (3) ***	10 (29) ***
*E*. *coli*	CspE	A 7-base long stretch of Us interrupted at a single position	this study	5 (5) ****	12 (55) ****

* 19 weak binders were analyzed and the number of those that contain the relevant CSP-recognition sites is shown. The total number of recognition sites in all the 19 mRNAs is shown in parenthesis

** 17 strong binders were analyzed and the number of those that contain the relevant CSP-recognition sites is shown. The total number of recognition sites in all the 17 mRNAs is shown in parenthesis.

*** Purine interruption at positions 2–5, excluding G at position 3.

**** Interruption at positions 2–6, excluding G at position 3 and purine at position 6

### Effect of CspE overexpression on membrane expression of IMPs

**Fig 11:**
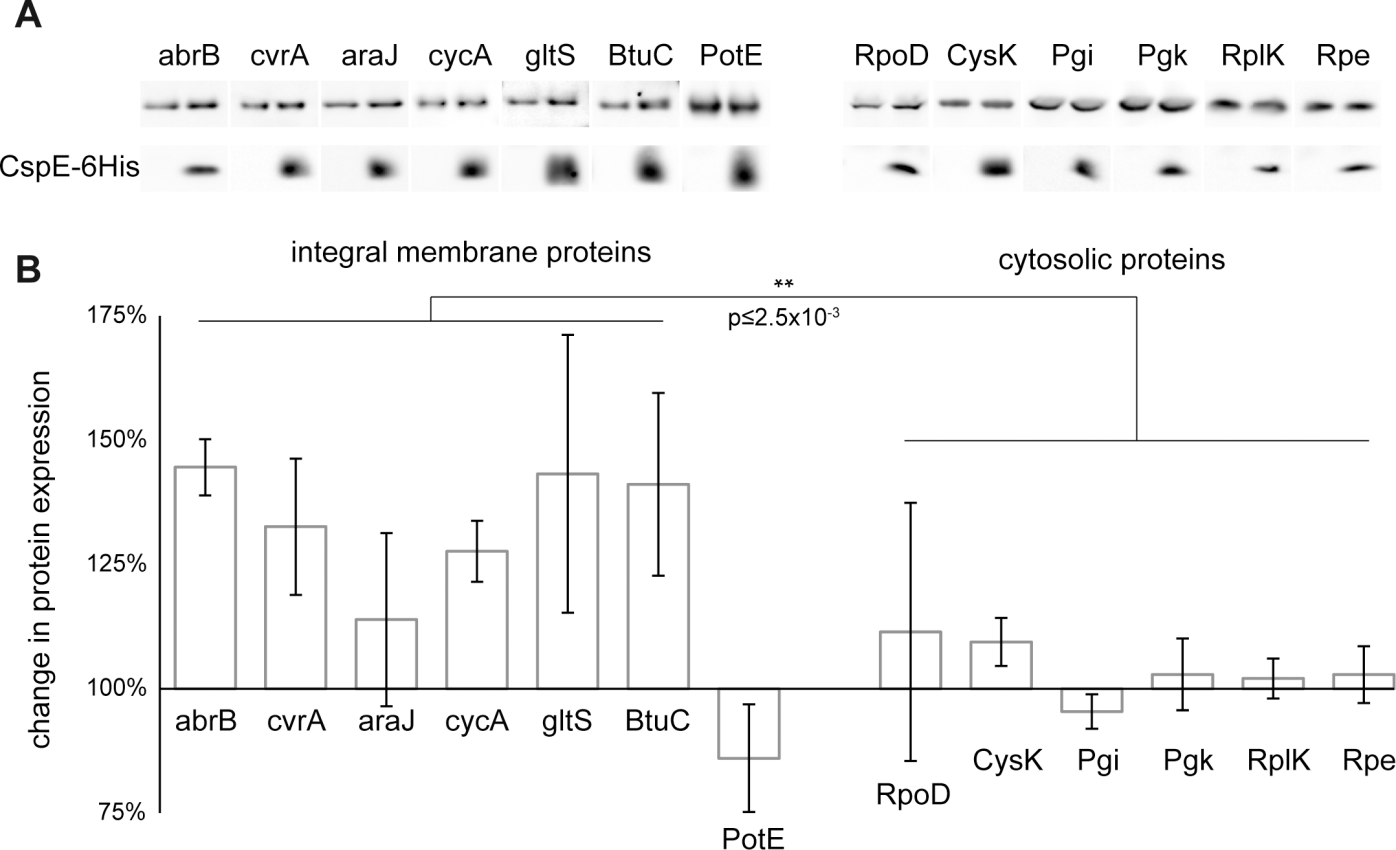
Expression of selected IMPs and cytosolic proteins: Effect of CspE-6H overexpression. The expression of several C-terminally 6His-tagged cytosolic proteins and IMPs was induced in cells that co-express CspE-6His or harbor an empty plasmid. Cell extracts (for cytosolic proteins) and floatation-purified membranes (for IMPs) were separated by tris-tricine PAGE, and analyzed by Western blotting **(A)**. The bands were quantified by densitometry (ImageJ, n≥3), and the average ratio (+ CspE /—CspE) is presented **(B)**. Error bars indicate SEM, Mann Whitney test was used to calculate p value.

How CspE influences the subcellular distribution of MPRs remains to be investigated. A possible scenario is that CspE and possibly also other CSPs serve as chaperones [35] specific for MPRs, during the early targeting stages that precede their association with membrane ribosomes. If this is true, it is expected that despite the fact that *E*. *coli* has 8 closely related CspE-homologues, overexpression of CspE-6His might have some effect on membrane protein biogenesis. To test this notion, we used wild-type *E*. *coli* harboring an empty plasmid or a plasmid encoding CspE-6H together with compatible plasmids encoding any of 7 selected IMPs or 5 cytosolic proteins, all of which carry a 6His-tag at their C-termini. The mRNAs of 5 of the selected IMPs (*abrB*, *cvrA*, *araJ*, *cycA*, and *gltS*) are strong CspE binders and their distribution to the ribosome-free fraction of the sucrose gradient is increased in cells overexpressing CspE-6His (Fig 10B). Each culture was disrupted by sonication, fractionated by flotation, and analyzed by SDS-PAGE and Western blotting (Fig 11A). The expression of cytosolic proteins was examined in total extracts, whereas the expression of IMPs was examined in the flotation-purified membranes (i.e. properly expressed). The results indicate that the expression of cytosolic proteins was generally similar in cells with or without overexpressed CspE-6H. In contrast, the expression of IMPs (except for PotE) was increased in membranes of cells co-expressing CspE-6H. A possible explanation for the exceptional effect of CspE overexpression on PotE would be that the *potE* gene transcription might be down-regulated by the cold shock protein, but this remains to be tested. Overall, since CspE positively affected both the localization of MPRs in the ribosome-free fraction and their expression as membrane integrated proteins, the results offer a putative functional linkage between the surprising subcellular distribution of MPRs and their translation into integral membrane proteins.

## Discussion

Previously, we hypothesized that MPRs reach membrane-associated ribosomes that were targeted to the membrane during the translation of the SRP-receptor FtsY [5, 20, 36] and showed that the FtsY-mediated ribosome targeting is feasible [37]. The proposed translation-independent MPR targeting to membrane-associated ribosomes implies that MPRs should bypass cytosolic ribosomes. In this paper, we initiated studies of this possibility by following the subcellular distribution of MPRs, using biochemical fractionations. Taken together, our results indicate that MPRs behave differently from CPRs in their subcellular distribution pattern and that unlike CPRs, the distribution of MPRs is specifically affected by overexpression of the cold shock protein CspE. We showed that MPRs are overrepresented in the membrane fraction and that overexpressed CspE-6His increased their membrane localization. Intriguingly, however, we also observed that MPRs were relatively enriched in the soluble, ribosome-free fraction and that the enrichment is more considerable under conditions of overexpressed CspE-6His. In contrast, the results suggest that compared with CPRs, MPRs are somewhat less associated with cytosolic ribosomes in cells overexpressing CspE-6His. These results suggest that there may be a cytosolic pool of MPRs that do not engage ribosomes, probably temporarily. Whether this reflects a step during their targeting to membrane-bound ribosomes remains to be investigated. The results also suggest that cold shock proteins, which express under normal physiological conditions (such as CspE), may play a role in the biogenesis of MPRs, possibly through their specific and direct interaction with this group of mRNAs [15] as RNA chaperones [38].

Concerning the role of the SRP-system in *E*. *coli* membrane protein biogenesis, we examined the effect of depletion of the SRP-receptor FtsY on the distribution of mRNAs to the membrane. The results show that under FtsY-depletion conditions the quantity of all mRNA subgroups is decreased on the membrane, suggesting that association of mRNAs with the membrane is, at least to some extent, dependent on the amount of membrane associated ribosomes, which is also decreased in these cells. Alternatively, mRNA targeting to the membrane might also require proper expression of membrane proteins that is inhibited in FtsY-depleted cells [23, 24]. Another possibility would be that depletion of FtsY leads to stress conditions, which cause impaired mRNA association with the membrane. Surprisingly, however, treatment with Kas, which also causes stress had little or no effect and on the amount of membrane associated mRNAs. One possible explanation would be that although sufficient for growth inhibition, a 30-min exposure to Kas might be too short for recycling ribosomes or membrane proteins that are stably associated with the membrane.

A crucial requirement for successful and meaningful mRNA distribution studies in *E*. *coli* by biochemical means is proper subcellular fractionation. Although our analyses of the various fractions generally confirmed their origin and relative purity, there are certain unknown aspects that might have affected the signal-to-noise ratio. First, and most importantly, the best RNA-seq data were obtained from the ribosome-free fractions. The reason for this is that the other fractions contain large amounts of ribosomal RNAs, making the identification of mRNAs in the RNA-seq less efficient. Nevertheless, we also observed meaningful differences between MPRs and CPRs in the ribosome-containing fractions, especially under conditions of CspE-6His expression. As mentioned in the Results section, the extent of other possible interferences remains unknown: (i) membrane-bound ribosomes might fall off the membrane during the centrifugation and/or (ii) cytosolic ribosomes might interact non-specifically with the membrane during the fractionation process. These obstacles could influence the apparent distribution of mRNAs to the ribosomal fraction and/or the membrane fraction. However, these elements of noisiness were apparently less significant in experiments with overexpressed CspE-6His, because it enhanced the differences between the subcellular distribution of MPRs and CPRs. In any case, the most important results were obtained by analyzing the ribosome-free fraction, where we do not anticipate any of the above concerns.

As mentioned, the most surprising observation was that MPRs are relatively enriched in the soluble, ribosome-free fraction, especially under CspE-6H overexpression. Future studies of the mechanistic reasons for this distribution pattern would be important because it may imply that: (i) transcription and translation of MPRs are largely uncoupled, as suggested recently [9], (ii) there should be a mechanism that decreases ribosome recruitment by MPRs in the cytosol, and (iii) the presence of MPRs in a ribosomal-free pool may represent an early stage during their targeting to the membrane.

The observation that MPRs and CPRs differ in their distribution patterns suggests that there should be a mechanism that distinguishes MPRs from CPRs. Our results have suggested that CspE and possibly also other CSPs [15] may specifically participate in the biogenesis of MPRs. CSPs bind DNA and RNA, and through this capacity they play several physiological roles [39, 40], in addition to their contribution to the cold shock response-network [33]. In certain cases CSPs are selective [41], and affect the expression of specific genes, as is the case with promoter-distal genes of the *metY*-*rpsO* operon [42], where CSPs have transcription antitermination activity. In other cases, however, CSPs seem to act as general mRNA chaperones [39]. Notably, many eukaryotic proteins have CSP-homologous domains (termed cold shock domains, CSD-proteins), several of them play essential pleotropic functions, such as the human Y-box-binding protein 1 (YB-1) [43]. Since MPRs usually have long uracil-rich stretches that encode trans-membrane helices [14], we suspected that CSPs might serve as specific MPR chaperones [15]. The results of the present work show that indeed, CspE-6His is associated with MPRs in the cytosolic, ribosome-free fraction. Interestingly, CspE-6His did not have any appreciable specific influence on the steady state amount of MPRs or CPRs (S5 Fig), suggesting that it might not play a general stabilizing role, as observed with other transcripts [44]. Instead, we revealed that CspE-6His overexpression has an appreciable specific effect on the subcellular distribution of MPRs. Whereas CspE-6His reduces the localization of MPRs in the cytosolic ribosomal fraction, it increases their distribution to the membrane and to the ribosome-free fractions. Since CspE is found almost exclusively in the ribosome-free fraction, we can conclude that its interaction with MPRs in that fraction is likely responsible for the observed changes in their overall distribution pattern.

In summary, our studies revealed unexpectedly that there is a pool of MPRs in the ribosome-free (cytosolic) fraction. We hypothesize that the MPRs enrichment in the ribosome-free fraction and on the membrane represents the beginning and the end of the targeting process, respectively. The questions which cellular factors prevent initiation of MPR translation in the cytosol and how, and which factors mediate their targeting to the membrane, as well as how CSPs act on MPRs remains to be investigated.

## Experimental procedures

### *E*. *coli* strains and plasmids

*E*. *coli* BW25113 [45] and its *ΔcspE*(kan):*ΔcspC* derivative [15] were used throughout this study. For FtsY-depletion experiments we utilized our lab strain, *E*. *coli* IY28 [27]. CspE-6His was expressed from pIE1-*cspE-6His* [15] and the pT7-5 vector was used as control. For co-expression of membrane or cytoplasmic proteins together with CspE, *abrB* was inserted by RF cloning into pZA32-luc [46] with unique AscI and ApaI sites and a C-terminal 6His tag coding sequence, instead of the luciferase gene (See S1 and S2 Tables). Then, all other genes were amplified by PCR with AscI and ApaI sites (See S1 and S2 Tables), digested with AscI and ApaI and ligated into similarly digested pZA32-*abrB*. All plasmids were verified by sequencing.

### Cell growth and expression conditions

Unless mentioned otherwise, colonies were inoculated for overnight growth at 37°C in LB medium, supplemented with ampicillin (100 μg/mL), kanamycin (30 μg/mL), or Cm (30 μg/mL), when necessary. Overnight cultures were diluted to an optical density (A_600_) of ~0.05 and grown in similar media and conditions. Expression of CspE-6His was induced by addition of 0.1% Arabinose at A_600_ ~ 0.2. In experiments for the investigation of mRNA expression and distribution, cells were harvested at A_600_ = 0.8–1.0. In co-expression studies with CspE, the expression of membrane or cytoplasmic proteins was induced 25 min after the induction of CspE-6His, by addition of 0.1 mM IPTG for 60 min. Arrest of translation was performed as described previously by addition of Cm and fast cooling [47]. Cm (100 μg/mL) was added to the culture at A_600_ = 1.0 and incubated for 10 min in the shaker at 37°C before cooling. For depletion of FtsY, overnight cultures of *E*. *coli* IY28 [27], were washed 3–5 times for removal of arabinose and diluted to A_600_ = 0.01–0.03 in LB medium with or without arabinose (0.1%). The cultures were incubated in the shaker at 37°C and harvested after 4 h. For treatment with Kas, overnight cultures were diluted to A_600_ = 0.01 in LB medium and incubated in the shaker at 37°C. Kas (1 mg/mL) was added after 3.5 h and the cultures were harvested after 4 h.

### Preparation of cell extracts

*E*. *coli* extracts were produced by sonication as described previously [24], with minor modifications. Cell pellets were washed with ice-cold 10% sucrose solution in 20 mM HEPES buffer (pH 7.5). Washed cells were suspended in extraction buffer (15 mM MgCl_2_, 20 mM KCI, 100 mM NH_4_CI, 20 mM HEPES, pH 7.5) to cell density of 40 A_600_ and supplemented with 0.2 mM β-mercaptoethanol, 5 U/mL RQ1 DNase (Promega), 250 U/mL RNaseIn-plus (Promega) and 1 mM pefabloc. Cells were sonicated on ice for 3x10 sec and cell debris was removed by centrifugation (10 min, 16,000g, 4°C). For FtsY- and Ffh-depletion experiments the extracts were produced differently in an attempt to minimize non-specific interactions between ribosomes and membranes. Cells were disrupted by 3 cycles of freeze and thaw followed by a mild and brief sonication (4x5 sec) and cell debris was removed by centrifugation (1 min, 16,000g, 4°C). The extraction buffer was modified by including 300 mM KAc instead of 100 mM NH_4_CI.

### Membrane floatation

Cell extracts were prepared as described above and ultracentrifuged at 260,000g for 1h. Each ultracentrifugation pellet was homogenized in 50 μL of ice-cold extraction buffer containing 0.15 M sucrose, and then mixed with 400 μL of extraction buffer containing 2.3 M sucrose. Two layers of sucrose buffers were gently added on top of the mixed solution (450 μL): 680 μL of extraction buffer containing 1.9 M sucrose and the 270 μL of sucrose-free extraction buffer. Membranes were purified by ultracentrifugation, as previously described [25]. The floated membrane ‘ring’ fraction was collected in a volume of 450 μL.

### Sucrose gradient ultracentrifugation

Cell extracts were prepared as described above and 60 μL or 450 μL of the extract were loaded on top of either a 1.3 mL 7.5%-25% (small scale) or a 12 mL (large scale), respectively of 7–22% or 10–30% linear sucrose gradient prepared in the same buffer. In large scale experiments the gradients were ultracentrifuged for 3.5–4 h (260,000g, SW41 rotor, Beckman centrifuge, at 4°C). In the small scale experiments the gradients were ultracentrifuged for 52 min (260,000g, TLS55 rotor, Beckman centrifuge, at 4°C). Fractions were collected (top to bottom) and the pellet was resuspended in a fraction volume of 7% sucrose solution. A_260_ was measured for each fraction using a NanoDrop spectrophotometer. Large scale fractions 1–5 (ribosome-free RNA) and fraction 14–17 (70S ribosomes) were pooled for further analyses (in the small-scale experiment fractions 1–3 and 7–9, respectively).

### His-tagged CspE pull-down assays from the ribosome-free sucrose gradient fraction

Top fractions 1–5 of the large-scale sucrose gradient were pooled, supplemented with 5 mM imidazole, and mixed with 0.15 mL of pre-equilibrated Talon beads. The beads were incubated with rotation for 30 min at 4°C, transferred to a column, washed once with 1.5 mL of 50 mM Tris pH 8, 150 mM NaCl, 5 mM imidazole and 2 more times with 1.5 mL of the same buffer containing 20 mM imidazole. His-tagged CspE and bound RNA were eluted with 250 mM imidazole, 300 mM NaCl, and 50 mM Tris pH 8.

### Western blotting

SDS-PAGE and Western blotting were performed as described [24]. We used the following polyclonal antibodies: rabbit anti-CspE antibodies [15]; anti-ribosomal protein antibodies from our lab collection; anti-SecE antibodies were obtained from Dr. Hajime Tokuda; goat anti-rabbit antibodies conjugated to horseradish peroxidase served as secondary antibodies (Jackson Immunoresearch). 6His-tagged proteins were detected by His-probe (Thermo-Fisher™).

### Northern blotting

Northern blotting was done with NorthernMax® Kit (Ambion™) as described in the kit protocol and blotted on BrightStar® Plus (Thermo-Fisher™) positively charged nylon membranes. RNA was detected with Biotin-labeled probes that were made with Biotin-16-dUTP (Roche). Probes were made with 25% Biotin-16-dUTP in PCR reaction as described in the protocol for Phusion high-fidelity DNA polymerase (Thermo) with primers that were described in S3 Table. Probes were then denatured for 5 min in 95 ^o^C and moved to ice-cold water. Denatured probes were incubated with Northern membrane overnight at 42 ^o^C and washed with NorthernMax stringency washes. The Blots were then incubated in Odyssey® Blocking Buffer (Licor) with 1% SDS for 1 h. Blots were then incubated with IRDye 800CW Streptavidin (Licor) for 1 h, washed 3 times in PBST, and exposed in the Licor Oddesy Fc imaging system.

### RNA extraction and semi-quantitative or quantitative PCR (qPCR)

RNA was extracted from 400 μL-samples with 400 μL of water-saturated Biophenol (tris-buffered phenol:chloroform:isoamyl alcohol 25:24:1). Mixtures were vortexed, incubated 10 min at room temperature and centrifuged (10 min, 12,000g at 4°C). 150 μL from the top aqueous phase were mixed with 150 μL of water-saturated chloroform. Mixtures were vortexed and centrifuged (10 min, 12,000g) and 70 μL of the top aqueous phase were mixed with 7.7 μL of 2 M sodium-acetate pH 5.3 and 196 μL of cold ethanol. Mixtures were vortexed, stored overnight at -80°C, and then centrifuged (15 min, 14,000 rpm, at 4°C). Supernatants were removed and the pellets were washed twice with 75% ethanol. The isolated RNA was dissolved in DEPC-treated water (15–50 μL) and the concentration was measured by NanoDrop. DNaseI treatment and removal was performed using DNA-free kit (AM1906, Ambion). cDNA was synthesized using a high capacity cDNA reverse transcription kit (Applied Biosystems). Semi-qunatitative PCR was done with Taq DNA Polymerase 2X Master Mix Red (Ampliqon) and 0.25 μM of each primer (S3 Table). For each cDNA sample a negative control was used that was not treated by reverse transcriptase. For each sample, 2.5 ng of template was added to PCR mix and semi-quantitative PCR was run for 30 cycles. PCR products were separated on 1% agarose gel with EtBr and imaged using UVIDOC 2HD UV camera. qPCR was performed using power SYBR green (Applied Biosystems), and an ABI 7300 or ViiA^TM^7 machine (qPCR primers are listed in S4 Table). RnpB and SsrA were used as endogenous controls. Ratios of fraction to extract concentrations were calculated for all mRNAs as 2^extract Ct^ / 2^fraction Ct^. Ct is cycle of threshold, which was 0.2 for all genes. PCR efficiency of all primers was verified by standard curves with -3 ≥slope≥ -3.6, R^2^>0.995.

### RNA-seq

Libraries were prepared as previously described [15]. Essentially, triplicate samples of rDNaseI treated RNA (1 μg) were fragmented at 70°C for 4 min (3 min for CspE-bound RNA, and none for RNA extracted from the top of the sucrose gradient), using RNA fragmentation kit (Ambion, AM8740). Fragmented RNA was purified using AMPure magnetic beads (Agencourt A63881) at a 2.2/1 ratio and reverse transcribed at a final volume of 12 μL. Second strand cDNA synthesis was performed by addition of the following: 3 μL NEB2 10X buffer, 1.2 μL dNTPs 10 mM, 1.2 μL dATP 10 mM, 0.8 μL *E*. *coli* DNA polymerase (NEB, M0209), 1.6 μL RNaseH (NEB, M0297), 0.4 μL T4 DNA ligase (NEB, M0202). Final volume was adjusted to 30 μL using DNase-free water and the reaction was incubated at 16°C for 2.5 h. Double strand cDNA was purified using magnetic beads, and undergone A-addition using KLENOW exo^-^ (NEB, M0212) in NEB buffer 2 supplemented by 167 μM dATP for 30 minutes at 37°C. Reaction product was purified using magnetic beads and ligated to adapters carrying the Illumina sequences using Quick ligation kit (NEB, M2200). The resulting libraries were amplified with 14 cycles of PCR using the PFUultraII fusion (Agilent). Libraries were sequenced by the INCPM center (Weizmann Institute of Science), on the Hiseq2500 and reads were mapped to the corresponding reference genome (NC_000913) using in-house scripts. The sequencing data were submitted to the National Center for Biotechnology Information Sequence Read Archive under Accession No. SRP063392.

### Expression of membrane and cytosolic proteins

*E*. *coli* BW25113 harboring a pZA32-based protein expression plasmid encoding the indicated membrane or cytosolic proteins, were transformed with either pIE1 (for CspE-6His expression) or an empty vector as control. Transformants were selected on LB-agar plates containing 10 μg/mL Cm and 100 μg/mL ampicillin. Overnight cultures were diluted and induced for protein expression and 50 A_600_ units of harvested cells were disrupted as described above. For analyzing expression of cytosolic proteins, extracts were separated by tris-tricine SDS-PAGE followed by Western blot analysis. For analyzing the steady state amount of integral membrane proteins, membranes were purified as described above (see Membrane floatation), prior to gel separation and Western blot analysis.

### Data analysis

Data analysis was performed using in-house Matlab scripts. Replicates were pooled after validation for consistence. Reads per kilobase per million mapped reads (RPKMs) were calculated for mRNAs (after removal of rRNA, tRNA and additional non-coding loci-mapped reads). MPRs were defined according to Uniport location SL-9909 (multi-pass membrane proteins) and modified by the removal of several genes annotated as outer-membrane and multipass. Cytoplasmic proteins were defined according to the PSORT database [48, 49] and modified by the removal of the *csps*. Functional annotation analysis with FDR-correction was performed using the DAVID functional annotation tool [50, 51]. Mann Whitney, and Kolmogorov-Smirnov (KS), and t tests were performed online using http://astatsa.com/WilcoxonTest/, http://www.physics.csbsju.edu/stats/KS-test.n.plot_form.html, and https://www.graphpad.com, respectively.

For clustering analysis, we selected 1596 genes that were detected in all the relevant libraries. Log2 values of the differential expression ratios in the various fractions were calculated and clustered into 5 clusters using the K-means algorithm (using Partek Genomic Suite Software). Then, a single-gene cluster was manually combined into the next most similar cluster. The distance used for the clustering was Euclidean dissimilarity. To determine the number of clusters, which well accommodate our data, we used the Davies-Bouldin index. Then, the number of clusters was chosen by manual inspection of local minima to ensure a good separation between clusters and cluster homogeneity.

## Supporting information

S1 Fig mRNAs distribution in additional sucrose gradient fractions and in flotation-purified membranes. For each fraction, as indicated within the figure, the RPKM ratio (fraction/total extract) of each mRNA was calculated. The average ratio of each group of mRNAs is presented as a % change from the average ratio of all detected mRNAs. This analysis was performed on data obtained from cells expressing CspE-6His (Fig 7A; fractions 1–3 = ribosome free, fractions 15–17 = polysomes, fractions 10–14 = 70S ribosomes). Error bars indicate SEM, n_MPRs_ = 460, n_CPRs_ = 986, n_all genes_ = 1862.
(PDF)

S2 Fig Identification of several mRNAs by PCR. Semi-quantitative PCR analysis of full-length transcripts in the ribosome-free fraction and whole cell extract. RNA without reverse transcriptase was used as a negative control.
(PDF)

S3 Fig Distribution of *all* MPRs and CPRs to the membrane fraction. *E*. *coli* extracts were fractionated by flotation through high-density sucrose for membrane purification. RNA was extracted from the total cell extract and the membrane fraction, and analyzed by RNA-seq (see **Fig 3**). The enrichment of all the detected mRNAs on the membrane was calculated as [RPKM_membrane_ / RPKM_extract_]. The quota of MPRs and CPRs in each 10^th^ percentile along the experimental landscapes, is presented as a moving average plot. (
(PDF)

S4 Fig Distribution of *all* MPRs and CPRs to the ribosome-free soluble fractions. *E*. *coli* extracts were fractionated by ultracentrifugation through a 7–22% sucrose gradient (see Fig 5). RNA was extracted from the total cell extract and from the pooled ribosome-free fractions of the gradient and analyzed by RNA-seq. The enrichment of all the detected mRNAs in the ribosome-free fractions was calculated as [RPKM_ribosome-free_ / RPKMe_xtract_]. The quota of MPRs and CPRs in each 10^th^ percentile along the experimental landscapes, is presented as a moving average plot.
(PDF)

S5 Fig Effect of CspE overexpression or of *cspE/C* deletion on mRNA levels does not correlate with their CspE-binding strengths. Total extract mRNA levels in CspE-6His overexpressing or *cspC*/*cspE*-deleted cells were determined by RNA-seq. The differential expression ratio of each detected mRNA was calculated by dividing its amount by its amount in wild type cells. The ratios were clustered by the K-means algorithm (Materials and Methods), which resulted in 5 groups of mRNAs (for example, the top cluster contains mRNAs that are increasingly abundant upon CspE-6His overexpression, and are reduced upon *cspE/C* deletion). After clustering, mRNAs within each cluster were sorted according to their CspE-binding coefficient (obtained by CspE-6H pull down assay), as color coded on the left column. Red: high CspE binding or increased expression; blue: low CspE binding or decreased expression.
(PDF)

S6 Fig Effect of cspACE deletion on the steady state mRNA levels of other csps. The mRNA level of the indicated genes was measured by qPCR in extracts of wild type *E*. *coli* and its isogenic ΔcspACE strain. The level of each mRNA was quantitated using specific primers, and the amount was normalized to a reference gene, rnpB, which is not related to the cold shock phenomenon. The experiment was repeated 3 times and error bars represent standard deviation.
(PDF)

S1 Table Cloning primers. (PDF)

S2 Table Plasmids. (PDF)

S3 Table Primers for semi-quantitative PCR. (PDF)

S4 Table qPCR primers. (PDF)

S5 Table Full list of all MPRs and CPRs that were identified in the RNA-seq of all the fractions and the cell extracts. (XLSX)

S6 Table List of the chosen set of MPRs and CPRs that were identified in the RNA-seq of each of the fractions. (XLSX)

S7 Table List of the chosen set of MPRs and CPRs that were identified in the RNA-seq of each of the fractions from cells overexpressing CspE-6H. (XLSX)

S8 Table Supplement to Table 1. Number of CSP-recognition elements in our defined MPR groups of strong and weak CspE binders.
(XLSX)
