# Supplementary figures and images for: Evidence for a cytoplasmic pool of ribosome-free mRNAs encoding inner membrane proteins in Escherichia coli
Source: PLoS One. 2017 Aug 25;12(8):e0183862. doi: 10.1371/journal.pone.0183862 (PMC5571963; doi:10.1371/journal.pone.0183862)

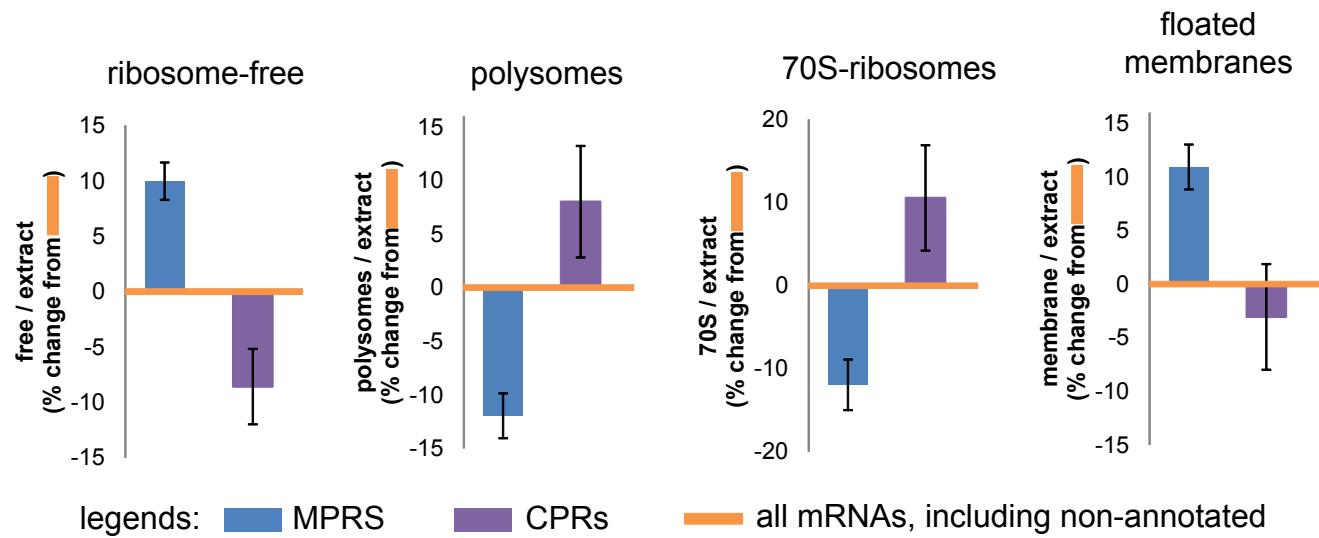

S1 Fig

Supplement: S1 Fig — For each fraction, as indicated within the figure, the RPKM ratio (fraction/total extract) of each mRNA was calculated. The average ratio of each group of mRNAs is presented as a % change from the average ratio of all detected mRNAs. This analysis was performed on data obtained from cells expressing CspE-6His (Fig 7A; fractions 1–3 = ribosome free, fractions 15–17 = polysomes, fractions 10–14 = 70S ribosomes). Error bars indicate SEM, nMPRs = 460, nCPRs = 986, nall genes = 1862. (PDF) [file pone.0183862.s001.pdf]

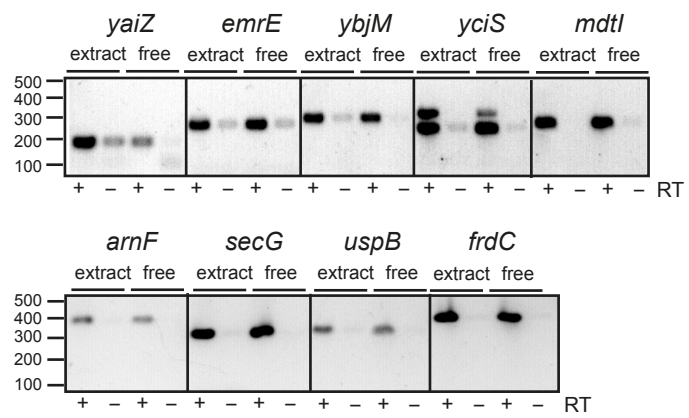

**S2 Fig**

Supplement: S2 Fig — Semi-quantitative PCR analysis of full-length transcripts in the ribosome-free fraction and whole cell extract. RNA without reverse transcriptase was used as a negative control. (PDF) [file pone.0183862.s002.pdf]

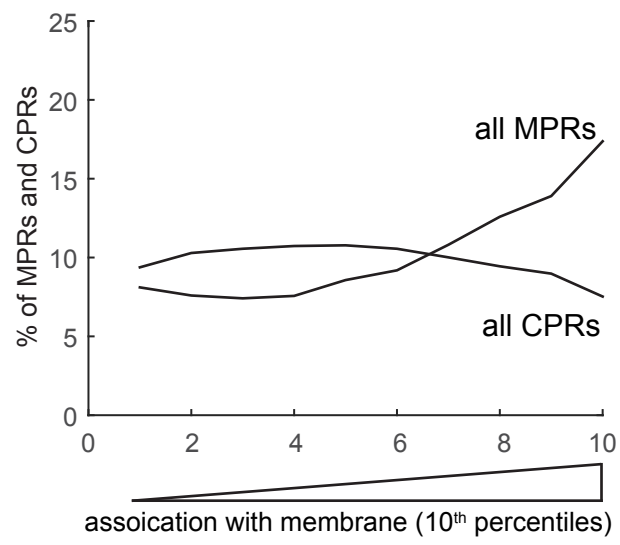

**S3 Fig**

Supplement: S3 Fig — E. coli extracts were fractionated by flotation through high-density sucrose for membrane purification. RNA was extracted from the total cell extract and the membrane fraction, and analyzed by RNA-seq (see Fig 3). The enrichment of all the detected mRNAs on the membrane was calculated as [RPKMmembrane / RPKMextract]. The quota of MPRs and CPRs in each 10th percentile along the experimental landscapes, is presented as a moving average plot. ( (PDF) [file pone.0183862.s003.pdf]

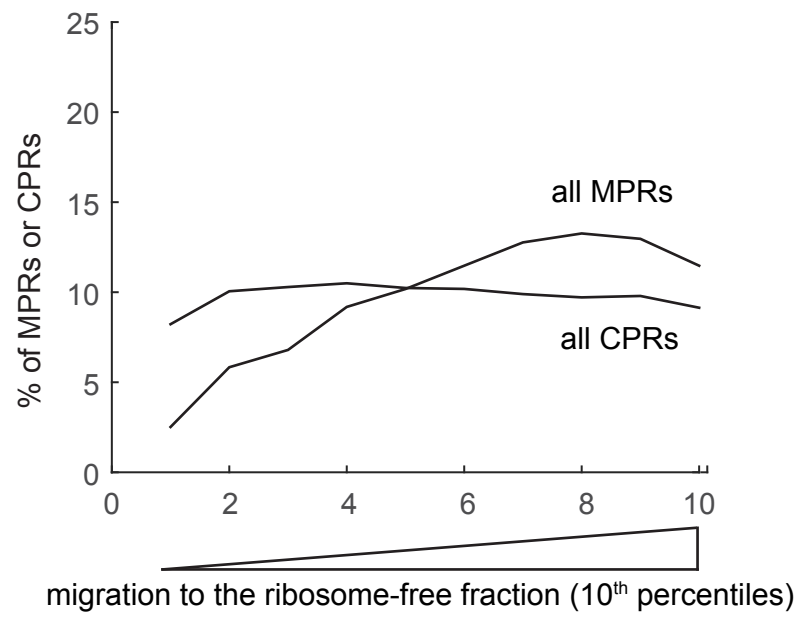

**S4 Fig**

Supplement: S4 Fig — E. coli extracts were fractionated by ultracentrifugation through a 7–22% sucrose gradient (see Fig 5). RNA was extracted from the total cell extract and from the pooled ribosome-free fractions of the gradient and analyzed by RNA-seq. The enrichment of all the detected mRNAs in the ribosome-free fractions was calculated as [RPKMribosome-free / RPKMextract]. The quota of MPRs and CPRs in each 10th percentile along the experimental landscapes, is presented as a moving average plot. (PDF) [file pone.0183862.s004.pdf]

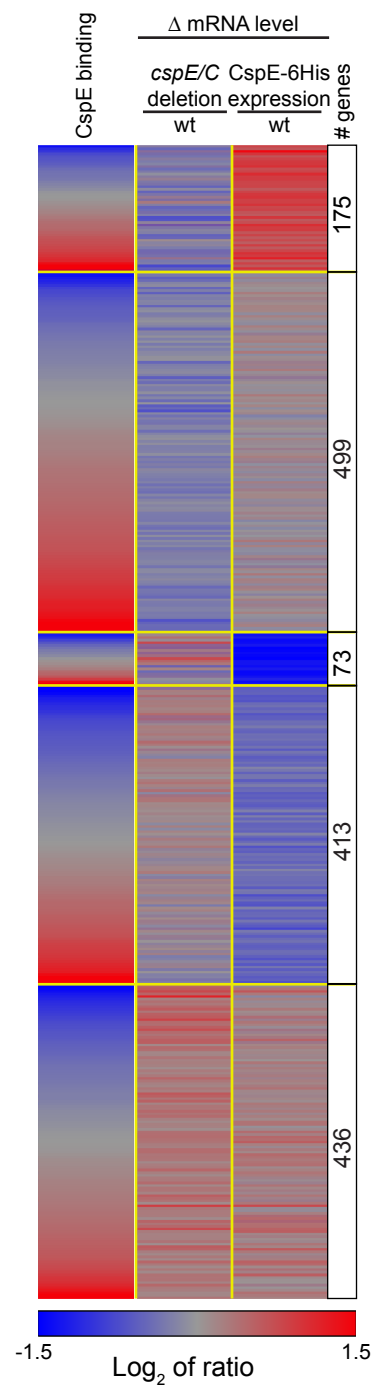

**S5 Fig**

Supplement: S5 Fig — Total extract mRNA levels in CspE-6His overexpressing or cspC/cspE-deleted cells were determined by RNA-seq. The differential expression ratio of each detected mRNA was calculated by dividing its amount by its amount in wild type cells. The ratios were clustered by the K-means algorithm (Materials and Methods), which resulted in 5 groups of mRNAs (for example, the top cluster contains mRNAs that are increasingly abundant upon CspE-6His overexpression, and are reduced upon cspE/C deletion). After clustering, mRNAs within each cluster were sorted according to their CspE-binding coefficient (obtained by CspE-6H pull down assay), as color coded on the left column. Red: high CspE binding or increased expression; blue: low CspE binding or decreased expression. (PDF) [file pone.0183862.s005.pdf]

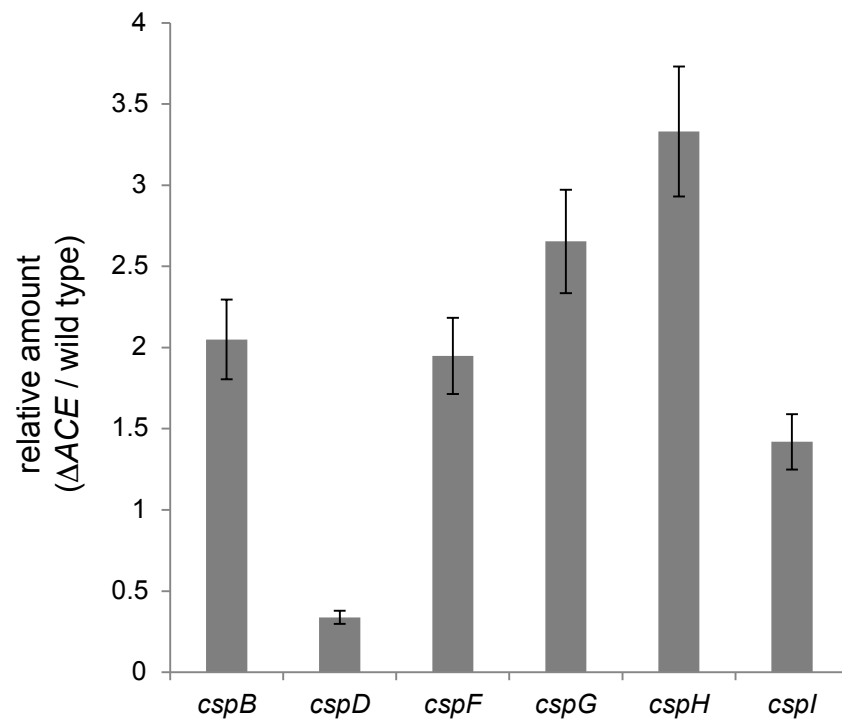

**S6 Fig**

Supplement: S6 Fig — The mRNA level of the indicated genes was measured by qPCR in extracts of wild type E. coli and its isogenic ΔcspACE strain. The level of each mRNA was quantitated using specific primers, and the amount was normalized to a reference gene, rnpB, which is not related to the cold shock phenomenon. The experiment was repeated 3 times and error bars represent standard deviation. (PDF) [file pone.0183862.s006.pdf]
