# Supplementary material for: Evidence for a cytoplasmic pool of ribosome-free mRNAs encoding inner membrane proteins in Escherichia coli
Source: PLoS One. 2017 Aug 25;12(8):e0183862. doi: 10.1371/journal.pone.0183862 (PMC5571963; doi:10.1371/journal.pone.0183862)
Supplement: S1 Table — (PDF) [file pone.0183862.s007.pdf]

Table S1. Cloning primers.

name	sequence (5' NNN…NNN 3')	use
abrB into pZA32_fwd	CCGAATTCATTAAAGAGGAGAAAGGGCCCATGCCAGTTTTGCAGTGGGGAATG	Insertion by RF of abrB-6His into pZA32-luc
abrB into pZA32_rev	CGTTTTATTTGATGCCTCTAGAGTCATTAATGGTGGTGGTGATGATGCGGCGCGCCGGCCGACCGCTTCGGCGCATAG	
cycA_fwd	catctaGGGCCCatggtagatcaggtaaaag	Insert gene between AscI and ApaI instead of abrB to pZA32(abrB-6His)
cycA_rev	agtaatGGCGCGCCtttccgcagttcagcag	
gltS_fwd	catctaGGGCCCatgtttcatctcgatactttag	
gltS_rev	agtaatGGCGCGCCaccggcaaaaatcgg	
araJ_fwd	catctaGGGCCCatgaaaaaagtcattttatc	
araJ_rev	agtaatGGCGCGCCcagtggtttcgccagc	
cvrA_fwd	catctaGGGCCCatggatgccacaacaataattag	
cvrA_rev	agtaatGGCGCGCCagattcagcttcctcttcag	
cysK_fwd	catctaGGGCCCATGAGTAAGATTTTTGAAGATAAC	
cysK_rev	agtaatGGCGCGCCCTGTTGCAATTCTTTCTCAG	
pgi_fwd	catctaGGGCCCATGAAAAACATCAATCCAACG	
pgi_rev	agtaatGGCGCGCCACCGCGCCACGCTTTATAG	
pgk_fwd	catctaGGGCCCATGTCTGTAATTAAGATGACC	
pgk_rev	agtaatGGCGCGCCCTTCTTAGCGCGCTCTTC	
rpe_fwd	catctaGGGCCCATGAAACAGTATTTGATTGCC	
rpe_rev	agtaatGGCGCGCCTTCATGACTTACCTTTGCC	
rplK_fwd	catctaGGGCCCATGGCTAAGAAAGTACAAGC	
rplK_rev	agtaatGGCGCGCCGTCCTCCACTACCAGG	
rpoD_fwd	catctaGGGCCCATGGAGCAAAACCCGCAG	
rpoD_rev	agtaatGGCGCGCCATCGTCCAGGAAGCTACG	
potE_fwd	catctaGGGCCCatgagtcaggctaaatcg	
potE_rev	agtaatGGCGCGCCaccgtgtttatttttcagttc	
btuC_fwd	catctaGGGCCCatgctgacacttgcccgcc	
btuC_rev	agtaatGGCGCGCCacgtcctgcttttaacaataac	
