# Supplementary material for: Evidence for a cytoplasmic pool of ribosome-free mRNAs encoding inner membrane proteins in Escherichia coli
Source: PLoS One. 2017 Aug 25;12(8):e0183862. doi: 10.1371/journal.pone.0183862 (PMC5571963; doi:10.1371/journal.pone.0183862)
Supplement: S2 Table — (PDF) [file pone.0183862.s008.pdf]

Table S2. Plasmids.

name	source	use / properties
pT7-5	[1]	Control vector
pIE1-*cspE-6H*	[2]	CspE-6H expression under araP
pZA32-luc	[3]	
pZA32(abrB-6His)		
pZA32(cvrA-6His)		
pZA32(araJ-6His)		
pZA32(cycA-6His)		
pZA32(gltS-6His)		
pZA32(btuC-6His)		
pZA32(potE-6His)	This study	Protein expression with varying CspE levels
pZA32(rpoD-6His)		
pZA32(cysK-6His)		
pZA32(pgi-6His)		
pZA32(pgk-6His)		
pZA32(rplK-6His)		
pZA32(rpe-6His)		

1. Bibi E, Kaback HR. In vivo expression of the lacY gene in two segments leads to functional lac permease. Proc Natl Acad Sci U S A. 1990;87(11):4325-9. Epub 1990/06/01. PubMed PMID: 2190220; PubMed Central PMCID: PMC54102.
2. Benhalevy D, Bochkareva ES, Biran I, Bibi E. Model Uracil-Rich RNAs and Membrane Protein mRNAs Interact Specifically with Cold Shock Proteins in Escherichia coli. PLoS One. 2015;10(7):e0134413. doi: 10.1371/journal.pone.0134413. PubMed PMID: 26225847; PubMed Central PMCID: PMCPMC4520561.
3. Lutz R, Bujard H. Independent and tight regulation of transcriptional units in Escherichia coli via the LacR/O, the TetR/O and AraC/I1-I2 regulatory elements. Nucleic acids research. 1997;25(6):1203-10. PubMed PMID: 9092630; PubMed Central PMCID: PMC146584.
