# Supplementary material for: Evidence for a cytoplasmic pool of ribosome-free mRNAs encoding inner membrane proteins in Escherichia coli
Source: PLoS One. 2017 Aug 25;12(8):e0183862. doi: 10.1371/journal.pone.0183862 (PMC5571963; doi:10.1371/journal.pone.0183862)
Supplement: S3 Table — (PDF) [file pone.0183862.s009.pdf]

Table S3. Primers for semi-quantitative PCR.

Name	Sequnece (5’ NNN….NNN 3’)
arnF_fwd	GATGTGGGGATTATTCAGCG
arnF_rev	CGTTGTTTTGTCGTGGGC
emrE_fwd	GGTGGTGCAATACTTGCAG
emrE_rev	TGTGGTGTGCTTCGTGAC
frdC_fwd	GTAAACCGTATGTACGGCC
frdC_rev	CCAGTACAGGGCAACAAAC
mdtI_fwd	GCGCAGTTTGAATGGGTTC
mdtI_rev	GGCAAGTTTCACCATGATCATTC
secG_fwd	CCTTATTGTGGCAATTGGCC
secG_rev	GGATATCGCTGGTCGGC
uspB_fwd	CCGTCGCATTATTTTGGGC
uspB_rev	CAATGCAATCAGGCTGACC
yaiZ_fwd	GCCTGTAAAAATCCGCCG
yaiZ_rev	CAGTTTCTTCATCGCGACG
ybjM_fwd	CATAAACAACGTTGGGCGG
ybjM_rev	CTGTGCTGTGGTTTAAACAAAC
yciS_fwd	TACTGGTGTTAGCGATCTTCG
yciS_rev	TTCCTTCGCCGCTGAC
