# Supplementary material for: Evidence for a cytoplasmic pool of ribosome-free mRNAs encoding inner membrane proteins in Escherichia coli
Source: PLoS One. 2017 Aug 25;12(8):e0183862. doi: 10.1371/journal.pone.0183862 (PMC5571963; doi:10.1371/journal.pone.0183862)
Supplement: S4 Table — (PDF) [file pone.0183862.s010.pdf]

Table S4. qPCR primers.

name	sequence (5' NNN…NNN 3')	Use
pgpB_fwd	TTCCGGTCACACGATGTTTG	MPRs
pgpB_rev	CCAGACCAGCAAGATAGCAATG	
cyoB_fwd	GTTACGATCGCTGGCATTATTTT	
cyoB_rev	CGGAGGTCAGCCACTCTTTC	
dppB_fwd	CCGATGTGGCAGCAGTATCTC	
dppB_rev	GCACGAACTCTTCCCAAACC	
mlaE_fwd	GCGTCGCTCGGACATAAAG	
mlaE_rev	GTTTGCGAAATTCCGGTTTG	
prfA_fwd	AGGAACGTTTTCGCGCATTA	CPRs
prfA_rev	GCGGTTTCGATATCTTCCTGAA	
rpoD_fwd	ATGGGCACCGTTGAACTGTT	
rpoD_rev	GATATTCAGCAACGGAGCATTG	
RnpB_fwd	CGTACCTTATGAACCCCTATTTGG	endogenous control
RnpB_rev	GGTGAAAGGGTGCGGTAAGA	
SsrA_fwd	TCTGGATTCGACGGGATTTG	
SsrA_rev	AGCGTAGTTTTCGTCGTTTGC	
